# Crosstalk between lactylation and other post-translational modifications in health and diseases

**DOI:** 10.1186/s43556-026-00500-w

**Published:** 2026-07-16

**Authors:** Wentao Qi, Jimin Su, Xiangming He, Fu Yang, Qinglin Li, Bowen Li

**Affiliations:** 1 The Department of Medical Genetics, Naval Medical University, Shanghai, China; 2 Department of Ultrasound, Hangzhou Geriatric Hospital, Affiliated Hangzhou First People’s Hospital Chengbei Campus, School of Medicine, Westlake University, Hangzhou, Zhejiang China; 3 Zhejiang Cancer Hospital, Hangzhou, Zhejiang China; 4 Postgraduate Training Base Alliance of Wenzhou Medical University (Zhejiang Cancer Hospital), Hangzhou, Zhejiang China; 5 Hangzhou Institute of Medicine (HIM), Chinese Academy of Sciences, Hangzhou, Zhejiang China; 6 Key Laboratory of Biosafety Defense, Ministry of Education, Shanghai, China; 7 Shanghai Key Laboratory of Medical Biodefense, Shanghai, China; 8 Tongde Hospital of Zhejiang Province, Hangzhou, Zhejiang China

**Keywords:** Lactylation, Post-translational modification, Crosstalk, Metabolic and Epigenetic regulation, Health and diseases

## Abstract

The inherent perception of lactate as the byproduct of glycolysis has been transformed by the discovery of lysine lactylation (Kla). Lactylation modification, a reversible and precise acylation, occurs on both histones and non-histone proteins and directly regulates epigenetics and the diversity of various protein functional activities. Since the discovery of lactylation, it has become evident that lactylation does not act in isolation but often works in conjunction with other post-translational modifications (PTMs) such as acetylation, phosphorylation and ubiquitination. On the one hand, lactylation modification shares the similar enzymes, modified sites and substrates with other acylation modifications, leading to functional crosstalk between them. On the other hand, lactylation or other PTMs could alter the protein conformations, amino acid side chain charges or the occupation of special sites, engaging in cooperative or competitive crosstalk, thereby influencing changes in their modification levels, signal transduction, conformation, re-localization and degradation. However, the underlying molecular mechanisms of the diverse crosstalk between lactylation and other PTMs, as well as their roles in various biological processes, have yet to be fully elucidated. Recent advances in quantitative mass spectrometry, isotope tracing, and integrative multi-omics have enabled more reliable detection and dynamic analysis of lactylation–PTM crosstalk, helping to distinguish causal regulation from metabolic correlation. This review integrates current mechanistic and functional evidence, highlighting the roles of lactylation crosstalk in pathophysiological processes and its implications for health and diseases.

## Introduction

Lactylation, a novel PTM, is characterized by covalent attachment of lactate at the special lysine residues of the proteins. It was initially discovered during bacterial infection, where lactylation participates in regulating the homeostasis of M1 macrophages [1]. Emerging evidence demonstrated that both histone and non-histone proteins can be modified with lactylation [2–11]. Like other epigenetic modifications, lactylation alters the chromatin structure and accessibility and regulates the expression of genes. For instance, the accumulation of histone H3 lysine 9 lactylation (H3K9la) and H3K18la in CD8^+^ T cells improved the chromatin accessibility resulting in increased expression of *Mitochondrial fission 1 (Fis1)* and *Optic atrophy 1 (Opa1)* [12]. Lactylation of the non-histone proteins significantly influences the transcriptional activity, enzyme activity and the stability of proteins [4–6, 13–16]. Lactylation of p53 at the K120 and K139 residues impaired its DNA-binding ability leading to inhibition of transcriptional activity and reducing p53 tumor-suppressing potential [17].

The crosstalk between proteins is as vital to biological functions as oxygen is to life, a regulatory dynamic largely orchestrated by diverse PTMs [15, 18–25]. Among these, lactylation has emerged as a key regulatory mechanism. Recent studies have revealed that lactylation does not function in isolation but exerts its pathophysiological effects through cooperative or competitive crosstalk with other PTMs (acetylation, phosphorylation, ubiquitination, methylation, SUMOylation and crotonylation), thereby modulating the balance between health and disease [26–31]. Under physiological conditions, lactylation participates in multiple beneficial biological processes, including energy metabolism (e.g., muscle homeostasis) [32], inflammatory responses (e.g., immune regulation) [33, 34], cell fate determination (e.g., stem cell maintenance and differentiation, meiosis) [35, 36], and development (e.g., neural development, neurite outgrowth and branching, and folliculogenesis) [37–39]. However, when this homeostatic balance is disrupted, dysregulated crosstalk can initiate or exacerbate various pathological conditions, such as inflammatory dysregulation, metabolic imbalance, and aberrant cell fate determination [1, 26, 40].

It is worth noting that the competitive or collaborative crosstalk between lactylation and other PTMs may result in different signal transduction and biological activities [26, 28, 41]. For instance, liver injury induced the accumulation of lactate and H3K18la in hepatic stellate cells. Pharmacological inhibition of class I histone deacetylases (HDACs) markedly elevated H3K18ac levels. H3K18la and H3K18ac compete for the same histone residue, and this antagonism ultimately downregulated the expression of multiple H3K18la-dependent metabolic genes, thereby delaying the progression of liver fibrosis [26]. Regarding collaboration, for non-histone proteins, lactylation could suppress ubiquitination by inhibiting the interaction between ubiquitin ligase and its substrates. Transcription factor EB (TFEB), a key transcription factor governing autophagy, undergoes lactylation at lysine 91. This modification impairs the interaction between TFEB and the ubiquitin ligase, thereby reducing its ubiquitination and proteasomal degradation and elevating TFEB protein levels [28]. Similarly, sustained inflammatory damage could induce vascular smooth muscle cells (VSMCs) to transdifferentiate into macrophage-like cells leading to vascular hyperplasia and atherosclerotic complications. Mechanistically, the PI3K-AKT signaling pathway phosphorylated the Sex-determining region Y (SRY)-related HMG-box gene 10 (Sox10), and then Sox10 undergo lactylation in a phosphorylation-dependent manner. The lactylation and phosphorylation of Sox10 act in a coordinated manner and contribute to transcriptional activation during VSMC transdifferentiation [41]. More importantly, lactylation shared the common enzymes and modification sites with other acylation modifications like acetylation and crotonylation which supplies more molecular foundations for the crosstalk [1, 42, 43]. Therefore, exploring the crosstalk between lactylation and other PTMs remains an essential frontier for studying the mechanisms of normal biological processes and diseases.

This review focuses on exploring the potential molecular mechanisms of lactylation in conjunction with other PTMs under both health and disease conditions, as well as assessing its viability as a potential therapeutic target. It also examines detection techniques for lactylated proteins and their potential roles in diagnosis and clinical application, aiming to bridge the gap between basic lactylation research and clinical applications by identifying promising targets and strategies for disease intervention.

## Molecular basis of lactylation

The long-standing view of lactate as a mere byproduct of glycolysis has been fundamentally revised by the discovery of lysine lactylation (Kla), a reversible post-translational modification involving the covalent attachment of a lactyl group to the ε-amino group of lysine residues [1]. Initially identified on histones, lactylation is now recognized as a widespread modification on both histone and non-histone proteins, regulating chromatin organization, transcription, enzymatic activity, protein stability, and subcellular localization [2, 4, 11, 15, 44]. Unlike classical epigenetic marks, lactylation directly links cellular metabolic status to protein function by translating intracellular lactate fluctuations into biological signals. Importantly, lactylation rarely functions independently; instead, it extensively intersects with other PTMs, including acetylation, phosphorylation, ubiquitination, methylation, SUMOylation, and crotonylation, through shared enzymes, overlapping modification sites, and coordinated regulatory networks [26–31]. Elucidating its molecular basis is therefore essential for understanding the dynamic regulation and functional outcomes of PTM crosstalk in both physiological and pathological contexts.

Chemically, lactate exists as L-, D-, and racemic DL-isomers, giving rise to three structurally related lysine modifications: L-lactylation (KL-La), D-lactylation (KD-La), and Nε-(carboxyethyl)-lysine (Kce) [15, 45–48]. Under conditions of elevated glycolysis, such as hypoxia or cancer metabolism, intracellular lactate accumulation predominantly favors KL-La formation, as L-lactate is the major physiological enantiomer [15, 47, 49–52]. The introduction of an L-lactyl group increases lysine polarity and hydrophilicity, thereby modulating protein conformation, interactions, and stability [4, 15]. In contrast, KD-La arises mainly through non-enzymatic pathways involving lactylated glutathione and is typically detected under conditions of impaired glycolytic enzyme activity, microbial metabolism, or metabolic disorders. Due to its distinct stereochemical configuration, KD-La may confer unique substrate or site selectivity [45]. In metabolically balanced states, Kce may represent a prevalent lactate-derived modification. By introducing an acidic carboxyethyl group, Kce markedly alters lysine charge properties, influencing enzyme activity, protein interactions, and intracellular localization, and has been linked to oxidative stress, inflammation, and metabolic regulation [50, 52].

Lactate availability constitutes the central metabolic determinant of protein lactylation. Cellular lactate pools are regulated by both endogenous production and extracellular transport, rendering lactylation highly sensitive to metabolic reprogramming [53–55]. Lactate is primarily produced from pyruvate through the action of lactate dehydrogenase (LDH) during glycolysis [46, 53]. Enhanced glycolytic flux, as observed in activated immune cells, rapidly proliferating cancer cells, and hypoxic tissues, leads to substantial lactate accumulation. In these contexts, lactate functions not only as an energy metabolite but also as a signaling molecule that directly influences epigenetic and post-translational regulatory programs. Beyond intracellular production, lactate can be transported across cellular membranes via Monocarboxylate transporters (MCTs), particularly MCT1 and MCT4 [53, 54, 56–58]. This transport enables lactate exchange between different cell populations, allowing lactylation to be regulated in a non-cell-autonomous manner [59]. Such metabolic coupling is especially prominent in tumors and inflamed tissues, where lactate-rich microenvironments profoundly reshape cellular behavior [58]. Perturbations in glycolysis, mitochondrial respiration, LDH activity, or lactate transport directly influence intracellular lactate levels and, consequently, lactylation abundance [53, 54, 58, 60]. Pharmacological or genetic interventions targeting these metabolic pathways have been shown to modulate lactylation levels, highlighting lactylation as a downstream effector of metabolic regulation [54, 61, 62].

Collectively, these lactylation-related modifications differentially reshape the chemical properties and spatial configuration of lysine residues. Their functional consequences depend on the protein substrates, modification sites, stereochemical forms, and cellular context, providing a molecular basis for the functional diversity of protein lactylation and its extensive crosstalk with other PTMs.

### Enzymatic and non-enzymatic mechanisms of lactylation

**Fig. 1:**
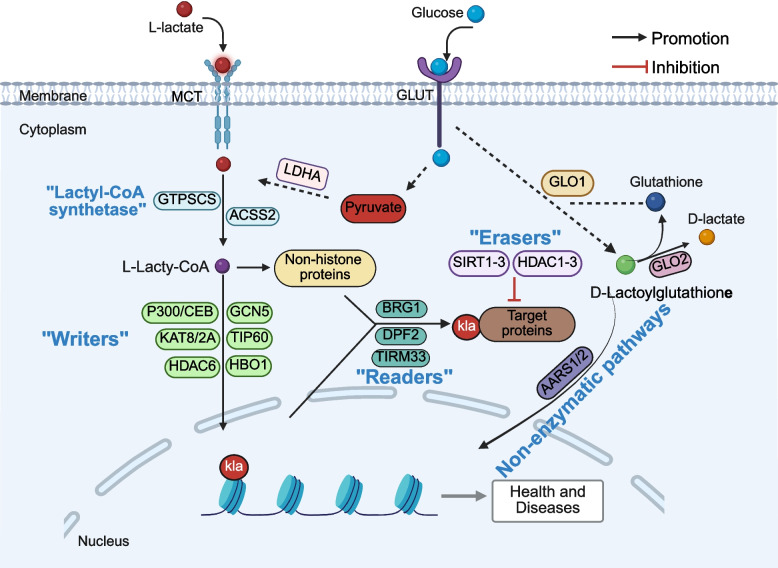
Molecular Basis of lactylation modification. This figure illustrates the molecular basis of lactylation modification, which is specifically categorized into two distinct modalities: the enzymatic pathway and the non-enzymatic pathway. In the enzymatic pathway, lactate is first converted to lactyl-CoA by lactyl-CoA synthetase; subsequently, lactylation modification is catalyzed by writer proteins following recognition by reader proteins, and the modified sites can be further reversed by erasers. In the non-enzymatic pathway, lactate directly acts as a substrate to mediate lactylation modification under other regulators such as AARS1/AARS2

Protein lactylation can arise through both enzymatic and non-enzymatic mechanisms (Fig. 1), which may operate concurrently depending on precursors, cellular context, metabolic status, and protein microenvironment [4, 8].

Enzymatic lactylation represents the best-characterized mechanism and is primarily mediated by acyltransferases that utilize lactyl donors to modify lysine residues. Accumulating evidence supports the existence of lactyl-CoA as a key activated intermediate that serves as the substrate for enzymatic lactylation. Lactyl-CoA can be synthesized by lactyl-CoA synthetases, including Acetyl-CoA synthetase 2 (ACSS2) and GTP-specific succinyl-CoA synthetase (GTPSCS), thereby providing the biochemical foundation for lactyl group transfer [63, 64]. Enzymatic Kla modification is mediated by specific “writer” enzymes that transfer lactyl groups to target proteins, altering their structure and function. These modifications are recognized by “reader” proteins to regulate downstream signaling. The process is dynamically reversed by “eraser” enzymes, with classical histone acetyltransferases playing key regulatory roles in this cycle [4, 8].

In addition to enzyme-catalyzed pathways, non-enzymatic lactylation has emerged as an alternative mechanism for protein modification, particularly under conditions of excessive lactate accumulation [48]. Unlike enzymatic Kla, non-enzymatic lactylation occurs through direct chemical reactions between lactate-derived intermediates and protein substrates, without the involvement of specific modifying enzymes, and is influenced by environmental factors such as pH, temperature, and metabolite concentration. This process is thought to become more prominent in settings of high glycolytic flux, including hypoxia, inflammation, and tumor microenvironments, where intracellular lactate levels are markedly elevated [8, 34].

Current evidence suggests that non-enzymatic lactylation primarily contributes to the formation of D-lactylation (KD-La) and Nε-(carboxyethyl)-lysine (Kce) [50, 52]. K_D-La is generated through reactions between proteins and S-D-lactylglutathione (LGSH), a metabolite produced by the glyoxalase pathway. Gaffney et al. demonstrated that cellular lactylation levels are regulated by LGSH availability and glyoxalase II (GLO2) activity, exemplified by modification at lysine 94 of phosphoglycerate kinase 1 (PGK1) [65]. In parallel, the highly reactive glycolytic byproduct methylglyoxal (MGO) can non-enzymatically modify multiple amino acid residues, including cysteine, arginine, and lysine; among these, lysine-directed reactions give rise to Kce, which has been detected in cells, albeit at lower abundance compared with MGO-derived arginine modifications [15].

Although the global contribution of non-enzymatic lactylation remains incompletely characterized, accumulating evidence supports its physiological relevance under metabolic stress conditions. Together, enzymatic and non-enzymatic lactylation pathways likely cooperate to shape the cellular lactylation landscape, enabling cells to dynamically adapt protein function in response to metabolic perturbations. Further elucidation of these regulatory mechanisms may reveal novel therapeutic opportunities targeting metabolic and inflammation-associated diseases.

### Writers, erasers, and readers of lactylation

Protein lactylation, as an emerging PTM, is dynamically regulated by a sophisticated enzymatic system comprising “writers” that install the modification, “erasers” that remove it, and “readers” that recognize the lactyl mark and transduce its biological signals [15]. The enzymatic addition of lactyl groups to lysine residues is catalyzed by a diverse array of transferases, whose activities are often context-dependent and may intersect with other PTM pathways, particularly acetylation (Kac) [8, 15].

Acetyltransferases as Multifunctional Writers: A key feature of Kla regulation is the involvement of classical acetyltransferases, which can utilize lactyl-CoA as an alternative donor substrate under specific metabolic conditions [4, 15]. For instance, E1A-binding protein p300/CREB-binding protein (p300/CBP), well-known histone acetyltransferases, have been demonstrated to act as lactyltransferases. They mediate Kla on transcription factors like Yin Yang 1 (YY1) [66]. Similarly, Tat-interactive protein 60 (TIP60) catalyzes lactylation of DNA repair protein Nijmegen breakage syndrome 1 (NBS1) and the autophagy regulator Vacuolar Protein Sorting 34 (VPS34), linking lactate flux to genome integrity and autophagic progression [32, 67]. Other acetyltransferases, including lysine acetyltransferases 8 (KAT8), p300/CBP-associated factor (PCAF) and Histone acetyltransferase binding to ORC1 (HBO1), have also been implicated in catalyzing both histone and non-histone lactylation, thereby regulating diverse processes from gene transcription to immune evasion [68–70].

The dual specificity of these enzymes for acetyl-CoA and lactyl-CoA raises questions about substrate preference in vivo. Catalytic selectivity is likely governed by multiple factors. From one perspective, while the acetyl group (CH₃CO-) fits precisely into conserved binding pockets, the larger, more polar lactyl group (CH₃CHOHCO-) may require specific accommodating residues, potentially explaining why some acetyltransferases show a relative preference [48, 71]. From another viewpoint, intracellular concentrations of acetyl-CoA are typically orders of magnitude higher than those of lactyl-CoA, which is present at trace levels. This disparity suggests acetyl-CoA is the dominant physiological substrate, with Kla occurring under conditions of locally elevated lactate and/or specific enzyme recruitment. Moreover, Definitive enzymatic constants (e.g., Km, kcat) for lactyl-CoA with proposed writers remain largely uncharacterized, making it difficult to assess catalytic efficiency and true in vivo relevance [15, 48, 71].

Beyond acetyltransferases, novel enzymatic pathways for Kla have been discovered. Notably, mitochondrial alanyl-tRNA synthetases (AARS1/AARS2) function as lactate sensors and lactyltransferases. Crucially, they utilize L-lactate and Adenosine Triphosphate (ATP) directly, without requiring lactyl-CoA as an intermediate, to modify a broad spectrum of targets including p53 and mitochondrial protein. This CoA-independent pathway represents a significant expansion of the lactylation toolkit [17, 34]. Furthermore, members of the Gcn5-related N-acetyltransferase (GNAT) family, such as General control nonderepressible 5 (GCN5) and YiaC, have also been identified as dedicated or promiscuous lactylases [72, 73]. Interestingly, even some deacetylases like Histone Deacetylase 6 (HDAC6) can exhibit lactyltransferase activity under high lactate conditions, competitively modifying α-tubulin at the same lysine residue targeted for acetylation, thereby linking metabolism to cytoskeleton dynamic [74].

The dynamic nature of Kla is underscored by the existence of specific “erasers”, primarily drawn from families of deacetylases, highlighting another layer of crosstalk with acetylation. Shared Erasers with acetylation: Histone deacetylases: Histone Deacetylase1-3 (HDAC1-3), HDAC8 and sirtuins: Sirtuin 1–3 (SIRT1-3) have been validated as effective delactylases [15]. For example, HDAC3 removes Kla from NBS1 and Apolipoprotein C-II (APOC2), modulating DNA repair and lipid metabolism, respectively [67, 75]. SIRT1 delactylates proteins like Canopy FGF Signaling Regulator 3 (CNPY3) to regulate cell death pathways in prostate cancer, while SIRT3 targets Cyclin E2 (CCNE2) to influence cell cycle progression in hepatocellular carcinoma [76, 77]. The reversibility conferred by these enzymes establishes Kla as a bona fide regulatory switch. The use of common erasers for Kla and Kac creates a direct molecular interface for competition. The modification state of a given lysine may thus be determined by the local balance between lactate/acetyl-CoA levels and the activity of bifunctional writers and erasers [48]. For instance, p300 and SIRT1 antagonistically regulate the lactylation status of α-Myosin Heavy Chain (α-MHC), a dynamic interplay critical for cardiac function [78].

The biological functions of Kla are ultimately mediated by reader proteins that specifically recognize the lactyl mark. Although this field is still evolving, initial readers are being identified. Emerging evidence points to chromatin remodelers and associated factors as among the first Kla readers. Proteins such as Brahma-related gene 1 (BRG1), D4, zinc and double PHD fingers family member 2 (DPF2), Double homeobox (Dux) and Tripartite Motif Containing 33 (TRIM33) can bind to lactylated histones, facilitating the recruitment of transcriptional complexes and altering chromatin accessibility. The systematic identification and characterization of a broader repertoire of readers, including those for non-histone targets, is a critical frontier for understanding how lactylation signals are translated into specific cellular responses [79–81].

In summary, the establishment and removal of Kla are mediated by a complex network of enzymes, many of which are shared or functionally intertwined with the acetylation machinery. This shared infrastructure—involving multifunctional acetyltransferases, common deacetylase erasers, and potentially overlapping readers—forms the core molecular basis for the extensive crosstalk observed between lactylation and other acyl modifications. The recent discovery of CoA-independent pathways (e.g., AARS1/2) adds further regulatory depth. Future work must quantitatively define enzyme kinetics in vivo, map context-specific writer/eraser/reader complexes, and elucidate how metabolic fluctuations precisely steer this enzymatic network to determine functional outcomes.

## Underlying mechanisms of crosstalk between lactylation and other PTMs

Since the first identification of lysine lactylation, accumulating studies have demonstrated that this novel PTM exerts critical roles in diverse pathophysiological processes. Lactylation tightly links the fields of epigenetics, metabolism, and post-translational regulation, serving as a key regulatory node across these disciplines [2, 7, 82–84]. With the deepening of related research, a growing body of literature has focused on the crosstalk between lactylation and other PTMs [19, 20, 85]. Therefore, this review systematically summarizes the research advances in the crosstalk between lactylation and other PTMs, as well as their underlying regulatory mechanisms.

**Fig. 2:**
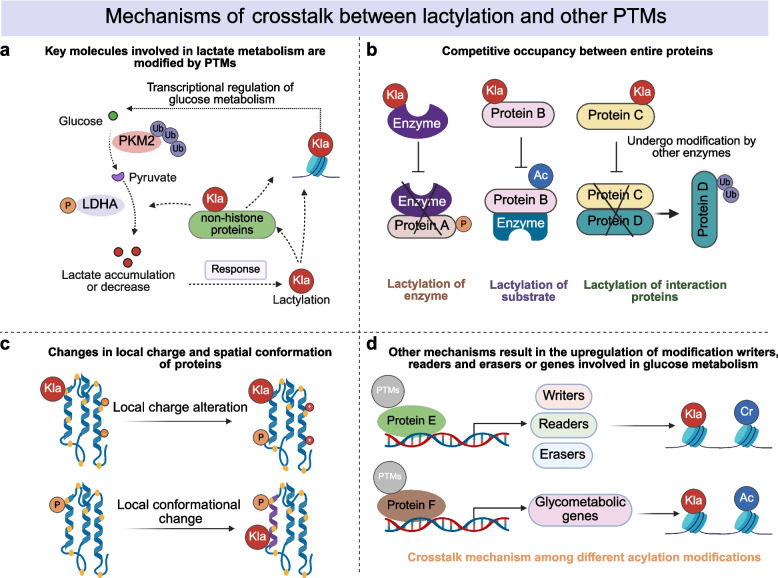
Underlying mechanisms of crosstalk between lactylation and other PTMs. **a** Schematic of crosstalk between protein lactylation and other PTMs in lactate metabolism. Non-lactylation PTMs of key metabolic enzymes regulate intracellular lactate abundance and global lactylation levels, driving the regulatory crosstalk between these modifications. **b** Lactylation of substrates, enzymes, or their interacting proteins alter or occupy steric hindrance, which perturbs protein PTMs and drives competitive crosstalk between these modifications. **c** One type of PTM (phosphorylation) induces changes in local charge or steric hindrance, thereby promoting the occurrence of another PTM (lactylation). **d** Transcription factor regulation affects the expression of lactate metabolism–related genes and acylation writers, readers, erasers, leading to potential crosstalk only among acylation modifications (acetylation & lactylation, acetylation & crotonylation)

The potential mechanisms underlying the crosstalk between lactylation and other post-translational modifications (PTMs) can be categorized as follows. The first mechanism refers to the regulation of lactate metabolism by other PTMs (Fig. 2a). Specifically, when key molecules involved in lactate metabolism are modified by other PTMs, lactate metabolism is enhanced, which in turn promotes protein lactylation and thereby enables crosstalk between different PTMs. For instance, phosphorylation-mediated activation of LDHA [32], ubiquitination and degradation of PKM2 [86], and phosphorylation-mediated activation of HK2 [26] can all trigger such crosstalk. Another mechanism is based on competitive occupancy between entire proteins (Fig. 2b). For example, lactylation of a substrate can prevent its ubiquitination by E3 ubiquitin ligases such as NEDD4 [87] or WWP2 [28]. An indirect form of competition also exists: lactylation of ALDH2 impairs its binding to the interacting protein PHB2, leading to increased free PHB2 levels and consequently promoting PHB2 ubiquitination [88]. The third mechanism involves changes in local charge and spatial conformation of proteins (Fig. 2c) [89, 90]. The occurrence of a preceding PTM alters the local charge and spatial structure of a protein, thereby facilitating a subsequent modification. For instance, CREB undergoes lactylation prior to phosphorylation [38], whereas Sox10 is phosphorylated before lactylation [41]; such sequential order is dictated by the conformational changes induced by the first modification [91]. Finally, an additional mechanism operates by upregulating the expression or activity of modifying enzymes or genes involved in glucose metabolism (Fig. 2d). This type of crosstalk is primarily observed among acylation modifications. For example, HDAC inhibitors block acylation erasers, thereby promoting the accumulation of both lactylation and crotonylation [31]. Tfap2 enhances p300 expression, which in turn increases histone lactylation and acetylation [92], and Glis1 upregulates glycolytic gene expression, leading to synergistic accumulation of histone lactylation and acetylation and subsequent chromatin remodeling [35]. Together, summarizing and uncovering these potential mechanisms will deepen our understanding of how lactylation and other PTMs function in health and disease, and may provide important implications for disease intervention and therapy.

## Crosstalk between lactylation and other PTMs in health

### Stemness and lineage commitment

#### Stemness maintenance

##### Lactylation and acetylation

**Fig. 3:**
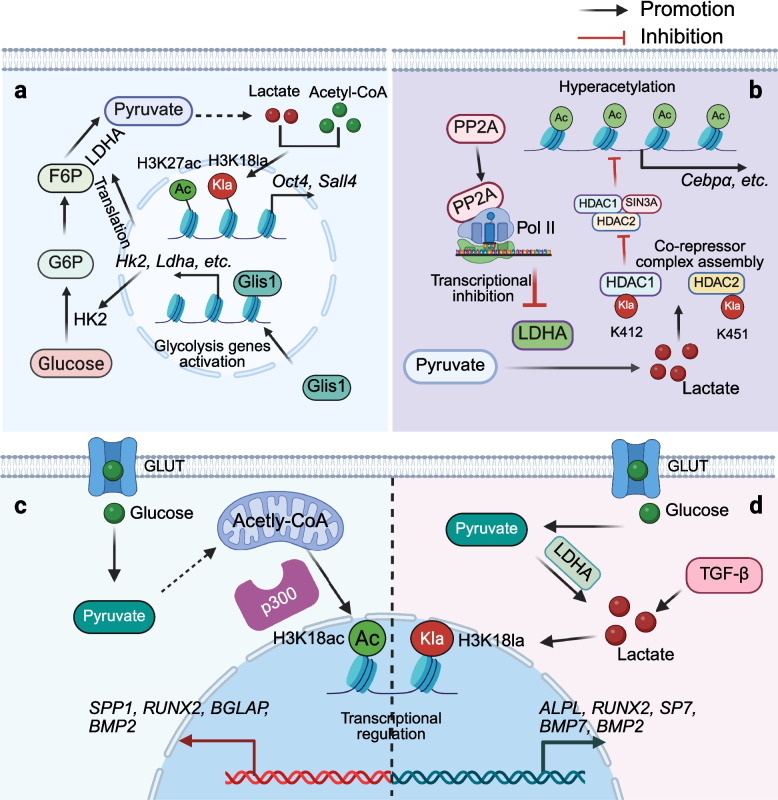
Crosstalk between lactylation and other PTMs in stemness maintenance. **a** Glis1 promotes the activation of glucose metabolism genes, leading to the accumulation of lactate and Acetyl-CoA, which in turn upregulates the levels of H3K18la and H3K27ac. H3K18la and H3K27ac remodel chromatin structure to drive the expression of *Otc4* and *Sall4*. **b** PP2A interacts with Pol II to maintain homeostatic LDHA expression, thus preventing HDAC1 and HDAC2 lactylation. The unlactylated HDAC1/2 assembles a SIN3A-containing corepressor complex to restrict histone hyperacetylation and preserve normal hematopoiesis. **c** Under physiological conditions, p300 utilizes substrate Acetyl-CoA to catalyze H3K18a. H3K18ac alters chromatin architecture, drives the expression of *SPP1, RUNX2, BGLAP and BMP2* and further promotes osteogenic differentiation. **d** Accumulation of H3K18la alters gene expression patterns, leading to aberrant hyperactivation of *ALPL, RUNX2, SP7, BMP7 and BMP2*, which in turn results in uncontrolled osteogenic differentiation

Epigenetic alterations are recognized as critical regulators of stem cell stemness. Histone acetylation and lactylation dynamics govern the maintenance of stem cell stemness including H3K27ac and H3K18la, which are interlinked during somatic cell reprogramming. The underlying mechanism involves Gli-similar 1 (Glis1) driving glycolytic reprogramming, increasing substrates for lactylation and acetylation, and leading to the concomitant accumulation of H3K18la and H3K27ac. Meanwhile, H3K27ac and H3K18la further synergistically boost the expression of cell reprogramming-associated genes (Fig. 3a) [35]. However, another study has reported contradictory conclusions. Li et al. have demonstrated that histone deacetylase sin3A-associated protein 30 (Sap30) specifically regulates key somatic genes and contributes to cellular reprogramming via the removal of H3K27ac [93]. These findings not only underscore the complexity of epigenetic regulation but also confirm that the crosstalk of H3K27ac and H3K18la is responsible for driving cellular reprogramming. In contrast, H3K27ac modification alone can inhibit cellular reprogramming.

#### Hematopoiesis

##### Lactylation and acetylation

Protein phosphatase 2 A (PP2A), a key regulator of hematopoiesis, is involved in the pathogenesis of multiple hematological disorders. Loss of PP2A stimulates the expression of lactate dehydrogenase A (LDHA) leading to the accumulation of intracellular lactate and lactylation. Mass spectrometry also detected lactylation of histone deacetylase 1 (HDAC1) at lysine residues 412 and HDAC2 at 451. Such modification impairs the assembly of the HDAC1/2/SIN3A co-repressor complex on chromatin, ultimately leading to increased histone acetylation. Excessive upregulation of histone acetylation drives dysregulated expression of hematopoiesis-associated genes, which in turn triggers aberrant hematopoiesis (Fig. 3b) [94]. The crosstalk between HDAC2 lactylation and histone acetylation sustains hematopoietic homeostasis. Consistently, LDHA inhibitors hold promising therapeutic potential for hematopoietic disorders driven by PP2A deficiency.

Of note, the authors did not assess the levels of histone lactylation. Accordingly, PP2A may regulate the crosstalk between histone lactylation and acetylation through multiple distinct mechanisms. First, lactate accumulation drives a synchronous increase in histone lactylation levels, and the two modifications may act in concert to reshape chromatin structure. Second, histone hyperacetylation exerts a dominant regulatory effect and inhibits the deposition of histone lactylation. This phenomenon may be attributed to the lower substrate concentration required for the initiation of acetylation. Taken together, the aforementioned unresolved questions must be further investigated in future studies to fully delineate the regulatory effects of PP2A on hematopoiesis.

#### Osteogenesis

##### Lactylation and acetylation

Histone lactylation and acetylation are involved in the regulation of osteogenesis. The level of H3K18ac is increased by p300 during osteogenic differentiation of adipose stem and progenitor cells (ASPCs), accompanied by upregulated expression of osteogenic differentiation genes *Bone morphogenetic protein 2 (BMP2), Runt-related transcription factor 2 (RUNX2), Bone gamma-carboxyglutamate protein (BGLAP)* and *Secreted phosphoprotein 1 (SPP1).* Meanwhile, p300 inhibitors significantly reduce the level of H3K18ac and osteogenic differentiation [95]. These results indicate that H3K18ac is essential for maintaining physiological osteogenic differentiation of ASPCs. However, under pathological conditions, H3K18la exhibits hyperlactylation and promotes abnormal osteogenesis by activating osteogenic genes *Alkaline phosphatase, liver/bone/kidney (ALPL), RUNX2, Bone morphogenetic protein 7 (BMP7), Sp7 transcription factor (SP7), Bone morphogenetic protein 2 (BMP2),* and *BGLAP* [96]*.* This indicates that H3K18ac and H3K18la regulate the activation of different osteogenic genes during osteogenic differentiation (Fig. 3c). H3K18ac is essential for maintaining physiological osteogenic differentiation, whereas hyper-H3K18la is a key factor contributing to dysregulated osteogenic differentiation.

Under pathophysiological conditions, H3K18ac and H3K18la regulate the expression of both shared target genes including *RUNX2* and *BMP7*, as well as distinct gene sets. This indicates that under pathophysiological conditions, these two modifications differ in both the repertoire and the magnitude of the gene expression they modulate. This discrepancy is largely determined by the effects of lactyl and acetyl moieties on the higher-order chromatin structure.

### Neurodevelopment and neural morphogenesis

#### Neural development

##### Lactylation and crotonylation

Histone crotonylation and lactylation are widely distributed in the brain and undergo global changes during neural development [37, 97, 98]. Histone H3 lysine 9 crotonylation (H3K9cr) and H3K18la are the main modifications that undergo changes and exert functions. Integrated genomic analyses, including genome-wide dynamics, Assay for Transposase-Accessible Chromatin using sequencing (ATAC-seq), and RNA-seq demonstrated that they function cooperatively to switch on distinct genes and regulate transcriptome remodeling during neural development. H3K9cr activates the expression of *Cytochrome c oxidase subunit 5 A (Cox5a), Succinate dehydrogenase complex subunit D (Sdhd),* and *Splicing factor 3a subunit 1 (Sf3a1),* while H3K18la activates the expression of *Raf-1 proto-oncogene, serine/threonine kinase (Raf1), BCL2 binding component 3 (Bbc3), and Cyclin dependent kinase inhibitor 1B (Cdkn1b)*. Furthermore, HDAC inhibitors can activate neuronal transcriptional programs by upregulating the levels of histone lactylation and crotonylation [31]. Although histone crotonylation and lactylation exert distinct effects on the remodeling of discrete chromatin regions and the regulation of target gene expression, their crosstalk has emerged as a key synergistic driver of neurodevelopment. Furthermore, treatment with HDAC inhibitors elevates both histone crotonylation and lactylation to promote neurodevelopment. This suggests that HDAC inhibitors suppress epigenetic erasers responsible for removing these acylation modifications, leading to the stable accumulation of crotonylation and lactylation.

#### Neurite outgrowth and branching

##### Lactylation and acetylation

Histone deacetylase 6 (HDAC6) acts as a lactyltransferase that utilizes lactate to catalyze the lactylation of α-tubulin, and this in turn enhances the dynamic assembly and disassembly of microtubules in neurons. Mass spectrometry identified a modification site at lysine K40 on α-tubulin, a residue where acetylation and lactylation undergo mutually exclusive competitive crosstalk. K40-lactylated α-tubulin promotes neurite outgrowth and branching in primary cultured hippocampal neurons by enhancing microtubule dynamics, whereas acetylation at K40 inhibits this process [74]. These findings indicate that the competitive crosstalk between acetylation and lactylation at the α-tubulin K40 locus plays a crucial role in regulating neurite morphogenesis of hippocampal neurons.

### Reproductive and tissue homeostasis

#### Folliculogenesis

##### Lactylation and phosphorylation

Follicle-stimulating hormone (FSH) serves as a key regulator of follicular development, mainly by promoting the proliferation and differentiation of granulosa cells (GCs) [99]. FSH activates the glycolytic pathway and elevates intracellular lactate levels. Elevated intracellular lactate levels promote the lactylation of lysine 136 in cAMP response element-binding protein (CREB), which subsequently contributes to the phosphorylation of its serine 133 residue. This dual modification facilitates the formation of the CREB/CBP/P300 complex and initiates transcriptional activation, thereby inducing the proliferation and differentiation of GCs. Furthermore, the sequential modification—lactylation preceding phosphorylation—is essential for the functional activation of CREB [38]. This reveals a precise crosstalk between lactylation and phosphorylation during folliculogenesis. A potential mechanism could be that lysine 136 and serine 133 of CREB are located within the same secondary structure of CREB. Lactylation alters the local protein charge distribution and conformation of CREB, thereby further promoting its phosphorylation.

#### Meiosis

##### Lactylation and acetylation

Transcription factor AP-2α (Tfap2a) is a critical sequence-specific DNA-binding protein that regulates the transcription of multiple genes by interacting with inducible viral and cellular enhancer elements [100]. Proper spindle assembly and faithful chromosome alignment during mouse oocyte meiosis require an appropriate level of Tfap2a. A previous study has demonstrated that Tfap2a upregulates the expression of p300. As a canonical writer enzyme for both lactylation and acetylation modifications, p300 subsequently elicits the concurrent increase of H4 lysine 12 acetylation (H4K12ac), H4 lysine 16 acetylation (H4K16ac), H3K18la and Histone H4 lysine 12 lactylation (H4K12la) which together orchestrate the meiotic process. However, overexpression of Tfap2a increases the levels of histone lactylation and acetylation, leading to abnormal spindles and misaligned chromosomes in mouse oocytes [92]. Tfap2a upregulates the expression of the writer p300, driving enhanced histone lactylation and acetylation. p300 mediates modification in a concentration-dependent, substrate-nonselective manner, which constitutes the molecular basis for the crosstalk between these two modifications.

#### Muscle homeostasis

##### Lactylation and phosphorylation

UNC-5-like kinase 1 (ULK1), a key regulator of muscle quality control, directly phosphorylates LDHA at serine 196, thereby enhancing its enzyme activity and promoting intracellular lactate accumulation. This metabolic shift subsequently induces lactylation of Vps34 at lysine residues 356 and 781. Vps34 lactylation, in turn, may facilitate autophagosome formation and maturation as well as endosome-lysosomal degradation processes essential for maintaining proteostasis and organelle turnover in muscle tissue [32, 101]. This suggests that the pathophysiological process underlying the phosphorylation and activation of LDHA may be accompanied by elevated lactate levels, enhanced lactylation modification, and close crosstalk between the two modifications.

## Crosstalk between lactylation and other PTMs in diseases

### Cancer

Distinct PTMs and their functional crosstalk weave a dense, interconnected and mutually reinforcing regulatory web across the cancer epigenome and proteome, acting in concert to fuel malignant transformation, tumor progression, therapeutic resistance, and metastatic dissemination [18, 21, 102, 103]. It is beyond dispute that lactylation, along with its crosstalk with other PTMs, exerts a non-redundant and critical regulatory role in these processes [2]. The biological basis for this is rooted in the fact that metabolic reprogramming, with the Warburg effect as its defining feature, is a core hallmark of malignant tumors [104–108]. Compellingly, widespread PTM dysregulation is a pervasive feature of cancer cells, including the hyperactivation of phospho-signaling cascades (most notably the AKT axis) [109], global alteration of histone methylation and acetylation [110], and aberrant ubiquitination of key immune evasion mediators exemplified by PD-L1 [111]. Accordingly, dissecting the multifaceted crosstalk between these PTMs holds great promise to deliver unanticipated anti-tumor efficacy, and more importantly, to identify novel synthetic lethal targets for cancer therapy. Here, we focus on reviewing the crosstalk between lactylation and other PTMs, summarize the underlying mechanisms of such crosstalk, and discuss their implications for exploring potential therapeutic strategies for different cancers.

#### Hepatocellular carcinoma

##### Lactylation and acetylation

**Fig. 4:**
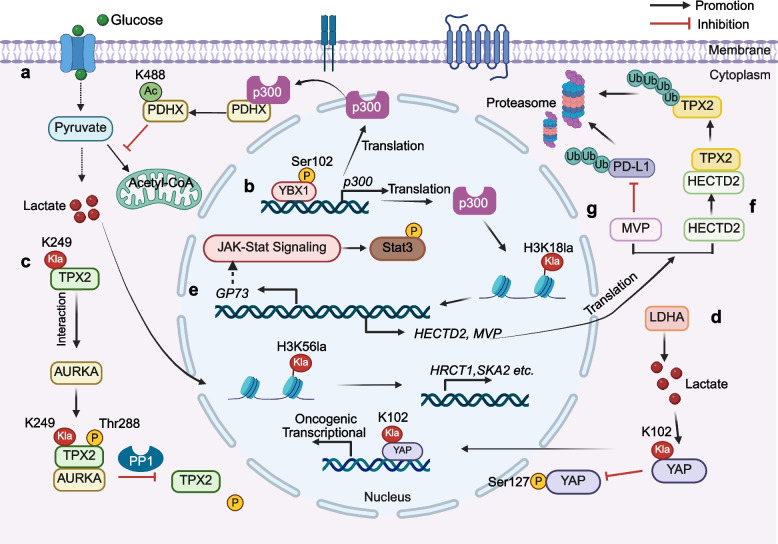
Crosstalk between lactylation and other PTMs in hepatocellular carcinoma. **a** p300-acetylated PDHX inhibits OXPHOS, boosts glycolysis and lactate accumulation to elevate H3K56la levels, which remodels chromatin to upregulate *HRCT1/SKA2* and drive HCC progression. **b** YBX1 phosphorylation activates its transcription to upregulate p300, which elevates H3K18la levels, remodels chromatin, enhances HECTD2/MVP expression, and drives chemoresistance. **c** TPX2 lactylation promotes its recognition and phosphorylation by AURKA, and concurrently inhibits PP1-dependent dephosphorylation of TPX2, ultimately accelerating cell-cycle progression. **d** YAP K102 lactylation inhibits Ser127 phosphorylation and boosts YAP oncogenic transcriptional activity. **e** H3K18la drives GP73 expression, activates JAK-STAT/STAT3 phosphorylation, and promotes tumor angiogenesis and metastasis. **f-g** H3K18la accumulation drives the upregulation of HECTD2 and MVP; HECTD2 promotes TPX2 ubiquitination and proteasomal degradation to confer lenvatinib resistance, whereas MVP blocks PD-L1 degradation to mediate HCC immune evasion

In hepatocellular carcinoma (HCC), the acetylation of pyruvate dehydrogenase complex component X (PDHX) is upregulated and closely associated with poor clinical prognosis. Mechanistically, acetylation of PDHX at lysine 488 weakens the interaction between PDHX and Dihydrolipoyl transacetylase (DLAT), thereby impairing the assembly and enzymatic activity of the pyruvate dehydrogenase complex (PDC). As a consequence, pyruvate flux into the tricarboxylic acid cycle is reduced, leading to enhanced lactate production, which fuels the Warburg effect and contributes to the increase of H3K56la (Fig. 4a) [112]. These metabolic (lactate accumulation) and epigenetic alterations (H3K56la) collectively reshape the transcriptional landscape of HCC and contribute to its progression.

##### Lactylation and phosphorylation

In HCC, Y-box binding protein 1 (YBX1) is frequently overexpressed and undergoes phosphorylation at serine 102, which contributes to its nuclear translocation. Nuclear YBX1 transcriptionally upregulates the histone acetyltransferase p300, resulting in increased H3K18la. In turn, H3K18la positively promotes *YBX1 and Yin-Yang 1 (YY1)* expression, ultimately inducing chemoresistance. The crosstalk between YBX phosphorylation and H3K18la establishes a reinforcing regulatory circuit linking metabolic reprogramming and epigenetic regulation (Fig. 4b) [113]. In parallel, lactylation of Targeting Protein for Xklp2 (TPX2) at lysine 249 disrupts its interaction with protein phosphatase 1 (PP1), sustaining its phosphorylation at threonine 288 by aurora kinase A (AURKA), ultimately accelerating cell-cycle progression (Fig. 4c) [114]. The Yes-associated protein (YAP) signaling pathway is constitutively activated and highly expressed in HCC. Numerous studies have demonstrated that the YAP signaling pathway is regulated by various PTMs, including lactylation and phosphorylation. Elevated lactate by LDHA further promotes lactylation of YAP at K102 while suppressing its phosphorylation at Ser127, collectively biasing YAP toward an oncogenic transcriptional state (Fig. 4d) [115]. Angiogenesis is an important cause of HCC progression. Another study reported that histone lactylation activates the transcription of golgi phosphoprotein 73 (GP73), which further accelerates the phosphorylation of STAT3 leading to a pro-angiogenic effect (Fig. 4e) [116]. Together, these findings underscore the coordinated contribution of lactylation–phosphorylation signaling to HCC progression and metastasis.

##### Lactylation and ubiquitination

Tumor cells often enhance their adaptive survival through dual mechanisms of therapy resistance and immune evasion [117]. For instance, H3K18la transcriptionally upregulates the E3 ligase HECT domain E3 ubiquitin protein ligase 2 (HECTD2), promoting ubiquitination-dependent degradation of Kelch-like ECH-associated protein 1 (KEAP1) and activating NRF2 antioxidant signaling, thereby conferring resistance to Lenvatinib (Fig. 4f) [118]. Concurrently, elevated H3K18la induces major vault protein (MVP) expression, stabilizing programmed death-ligand 1 (PD-L1) by preventing β-TrCP-mediated proteasomal degradation, which contributes to immune checkpoint resistance. Pharmacological inhibition of lactylation disrupts the crosstalk between lactylation and ubiquitination, thereby reversing the adaptive survival of HCC (Fig. 4g) [119].

#### Pancreatic cancer

##### Lactylation and acetylation

In pancreatic cancer, the Warburg effect leads to elevated lactate levels [120]. Lactylome profiling reveals a marked increase in global pan-Kla levels, among which H3K18la exhibits the most significant elevation. H3K18la transcriptionally activates acetyl-CoA acetyltransferase 2 (ACAT2), and ACAT2-mediated acetylation of mitochondrial carrier homolog 2 (MTCH2) enhances MTCH2 protein stability, promoting a metabolic shift from oxidative phosphorylation to glycolysis. This Metabolic reprogramming drives a further elevation of intracellular lactate, thereby establishing a positive feedback loop that sustains tumor cell proliferation. Notably, proteolysis-targeting chimera (PROTAC) strategies targeting ACAT2 have demonstrated therapeutic efficacy, highlighting the translational potential of disrupting lactylation–acetylation circuits [5].

##### Lactylation and phosphorylation

Pancreatic ductal adenocarcinoma (PDAC) is the most common and most malignant type of pancreatic cancer [121]. The crosstalk between lactylation and phosphorylation forms a positive loop to promote the progression of PDAC. For instance, threonine/tyrosine protein kinase (TTK) phosphorylates LDHA at tyrosine 239, enhancing its enzymatic activity and elevating intracellular lactate levels. Increased lactate contributes to H3K18la accumulation, which in turn augments TTK transcriptional activity, forming a self-amplifying loop that facilitates PDAC progression [122].

##### Lactylation and ubiquitination

Elevated TFEB lactylation was detected in clinical specimens of human pancreatic cancer. Under metabolic stress, lactate-mediated lactylation of TFEB represents a key adaptive survival strategy by sustaining autophagy–lysosomal capacity. Mechanistically, lactate contributes to autophagy and lysosome biogenesis by lactylating TFEB at lysine 91, which blocks WW Domain Containing E3 Ubiquitin Protein Ligase 2 (WWP2)-mediated ubiquitination and proteasomal degradation, resulting in TFEB accumulation and enhanced expression of autophagy–lysosomal genes. TFEB lactylation is implicated in proliferative signaling in pancreatic cancer, underscoring a crucial role of crosstalk between lactylation and ubiquitination in pancreatic cancer treatment [28].

#### Breast cancer

##### Lactylation and phosphorylation

**Fig. 5:**
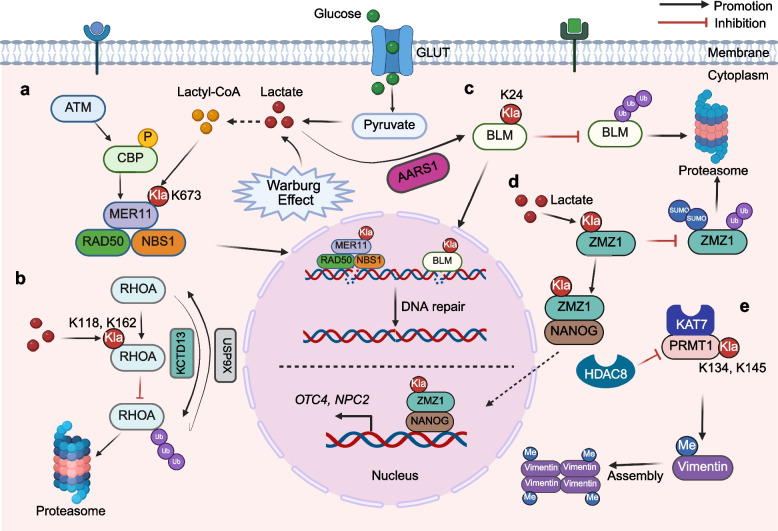
Crosstalk between lactylation and other PTMs in breast cancer. **a** ATM phosphorylates and activates CBP, which in turn lactylates MRE11 to promote the assembly of the MRE11-RAD50-NBS1 (MRN) complex, thereby facilitating DNA damage repair and ultimately conferring chemoresistance. **b** ROHA lactylation inhibits its ubiquitin-proteasomal degradation to sustain its oncogenic activity. **c** AARS1-mediated lactylation activates BLM and inhibits its ubiquitin-proteasomal degradation to enhance DNA damage repair and drive tumor chemoresistance. **d** ZMZI lactylation inhibits its ubiquitin-proteasomal degradation and enhances its binding to NANOG; the ZMZI-NANOG binary complex undergoes nuclear translocation to upregulate OTC4 and NPC2 expression, ultimately driving tamoxifen resistance. **e** PRMT1 lactylation enhances its methyltransferase activity to drive the methylation of Vimentin and the assembly of the corresponding quaternary complex

Radiotherapy and chemotherapy are the main therapeutic approaches for breast cancer [123]. A previous study found a significant positive correlation between intracellular lactate levels and the homologous recombination (HR) score, suggesting that lactylation may be involved in regulating the HR process. Mechanistically, ataxia-telangiectasia mutated kinase (ATM) phosphorylates and activates CBP, the writer enzyme that mediates Meiotic Recombination 11 Homolog 1 (MRE11) lactylation. Activated CBP in turn catalyzes the lactylation of MRE11 at lysine 673, which enhances the DNA-binding capacity of MRE11 and consequently promotes HR repair. Encouragingly, researchers successfully restored therapeutic sensitivity via the combined administration of a CBP lactylation inhibitor and LDHi, providing novel insights for breast cancer treatment (Fig. 5a) [27].

##### Lactylation and ubiquitination/SUMOylation

In Ras homolog family member A (RHOA)-driven cancers, lactylation at the oncogenic hotspots K118 and K162 counteracts KCTD13-mediated ubiquitination and degradation. Moreover, a process further reinforced by ubiquitin-specific protease 9X (USP9X). USP9X deubiquitinates RHOA, thereby further promoting the stability of RHOA. These findings highlight a cooperative lactylation–ubiquitination axis governing oncogenic signaling intensity (Fig. 5b) [124]. Similarly, in chemotherapy-treated cells, metabolic stress enhances AARS1-mediated hyperlactylation of Bloom syndrome protein (BLM) at lysine 24, blocking Mindbomb E3 ubiquitin protein ligase 1 (MIB1)-dependent ubiquitination and strengthening homologous recombination repair. Mutation of the lactylation site compromises DNA repair and restores chemosensitivity, underscoring the crosstalk between lactylation and ubiquitination as a key determinant of therapy resistance (Fig. 5c) [125]. Tamoxifen is a first-line therapy for Estrogen receptor (ER)-positive breast cancer and is frequently associated with the risk of drug resistance [126]. In tamoxifen-resistant tumors, glycolysis-associated lactylation of zinc finger MIZ domain-containing protein 1 (ZMIZ1) prevents SUMOylation and ubiquitination-mediated degradation, activates Homeobox protein NANOG transcriptional activity, and is implicated in endocrine resistance (Fig. 5d) [127].

##### Lactylation and methylation

Under hypoxic conditions, histone deacetylase 8 (HDAC8), a delactylase specifically targeting protein arginine methyltransferase 1 (PRMT1), is downregulated, thereby enhancing PRMT1 lactylation at lysines 134 and 145. Lactylated PRMT1 exhibits increased methyltransferase activity, catalyzing asymmetric dimethylation of vimentin at arginine 64 (R64) and ultimately promoting metastasis in triple-negative breast cancer (Fig. 5e) [128]. Therefore, targeted inhibition of PRMT1 lactylation is a promising therapeutic strategy for the treatment of triple-negative breast cancer.

#### Glioblastoma

##### Lactylation and acetylation

Glioblastoma, the most malignant primary tumor of the central nervous system, exhibits complex metabolic reprogramming and epigenetic alterations [129]. For instance, succinyl‑CoA ligase GDP‑forming subunit beta (SUCLG2) synergistically promotes glioblastoma progression through the above metabolic and epigenetic programs. First, SUCLG2 physically interacts with Lamin A (LMNA) and catalyzes the acetylation of LMNA at lysine 470, which impairs mitochondrial oxidative phosphorylation and induces mitochondrial dysfunction. Second, SUCLG2 directly binds to DLAT, thereby blocking the recruitment of lactate‑dependent H4K16la to the promoter regions and cis‑regulatory elements of key tumor suppressors. This inhibits the transcription of the tumor suppressor genes *Bestrophin 1 (BEST1), GRAM domain containing 4 (GRAMD4), and Methyl-CpG binding domain protein 6 (MBD6),* ultimately tipping the balance between cell proliferation and apoptosis to drive the malignant progression of glioblastoma [130].

GTPSCS is a lactyl-CoA synthetase that catalyzes the conversion of L-lactate to lactyl-coenzyme A, thereby providing the substrate for protein lactylation. Since histone modifications occur in the nucleus, acetylation of lysine 73 in the G2 subunit is required for GTPSCS nuclear translocation. Following nuclear entry, GTPSCS interacts with p300 to selectively elevate H3K18la, which may contribute to the expression of *Growth differentiation factor 15 (GDF15)* and ultimately promote cell proliferation and radioresistance [64]. In parallel, SIRT7-mediated deacetylation of H3K18 also contributes to malignant transformation and cell proliferation. H3K18 deacetylation may provide more opportunities for lactylation [131]. Therefore, H3K18 deacetylation and lactylation may act as parallel and complementary epigenetic events that jointly support oncogenic growth.

##### Lactylation and phosphorylation

In malignant glioma, the Warburg effect leads to excessive lactate accumulation and elevated histone lactylation [132]. This increase of histone lactylation induces the transcription of the NF-κB–associated long noncoding RNA-LINC01127, which subsequently enhances phosphorylation and activation of the JNK signaling pathway. Activated JNK signaling further reinforces glycolytic metabolism, forming a self-sustaining lactylation, LINC01127, phosphorylation feedback loop that supports glioma self-renewal and proliferation [133]. Moreover, the functional peptide P4-135aa, encoded by MAPK6P4, phosphorylates kruppel-like factor 5 (KLF15) at serine 238, enhancing its stability and nuclear localization. Activated KLF15 upregulates LDHA transcription, leading to increased lactate production and elevated lactylation of Vascular Endothelial Growth Factor Receptor 2 (VEGFR2) and Vascular endothelial cadherin (VE-cadherin), contributing to resistance to chemotherapy and radiotherapy [134]. The crosstalk between lactylation and phosphorylation provides new insights into the occurrence, development and therapy of glioma.

#### Lung cancer

##### Lactylation and phosphorylation

Immunotherapy is an important strategy for lung adenocarcinoma treatment, and lactylation is extensively involved in immune modulation [135]. Lactylation of cyclic GMP–AMP synthase (cGAS) at lysine 21 contributes to its nuclear export and ubiquitin-independent proteasomal degradation, suppressing interferon signaling. Conversely, phosphorylation of proteasome subunit alpha type 4 (PSMA4) at serine 188 disrupts PSMA4–cGAS interactions, counteracting cGAS degradation. Interestingly, lactylation of cGAS at K415 further modulates phosphatidylinositol-4,5-bisphosphate 3-kinase catalytic subunit beta (PIK3CB) activity and interferes with ULK1-mediated PSMA4 phosphorylation, reinforcing cGAS destabilization [136]. Through these coordinated mechanisms, lactylation–phosphorylation-ubiquitination crosstalk contributes to immune escape by degrading cGAS.

##### Lactylation and ubiquitination

In lung adenocarcinoma, AARS1 catalyzes lactylation of SUMO2 at lysine 11 (SUMO2-K11la), disrupting its interaction with long-chain acyl-CoA synthetase 4 (ACSL4). This could accelerate ACSL4 degradation, perturb lipid metabolic homeostasis, and enable tumor cells to evade ferroptosis. Competitive inhibition of the lactylation of SUMO2 by a cell-penetrating peptide restores ferroptotic cell death and sensitizes tumors to cisplatin, highlighting lactylation-dependent ubiquitination as a druggable vulnerability [30].

In non-small cell lung cancer, intracellular lactate accumulation leads to an increase in H3K18la. H3K18la contributes to keratin 19 (KRT19) transcription, which enhances myosin heavy chain 9 (MYH9)-dependent ubiquitination of p21, and inhibits p53-dependent *cyclin-dependent kinase inhibitor 1 A (CDKN1A)* activation. This could further retard cellular senescence and suppress CD8⁺ T-cell cytotoxicity. KRT19 depletion can synergize effectively with PD-1 blockade to inhibit tumor growth, suggesting that KRT19 is a promising therapeutic target [137].

#### Colorectal cancer

##### Lactylation and phosphorylation

Non-homologous end-joining (NHEJ) repair is a critical survival mechanism by which tumor cells evade chemotherapy and radiotherapy [138]. Phosphorylation–lactylation crosstalk also plays a critical role in DNA damage repair and chemoresistance. Upon DNA damage, activation of ATM induces phosphorylation of GCN5, strengthening its interaction with XRCC4-like factor (XLF) and facilitating lactylation of XLF at lysine 288. Lactylated-XLF cooperates with K70 to promote the efficiency of NHEJ, enabling tumor cells to evade radiotherapy and chemotherapy [139].

##### Lactylation and ubiquitination

Consistent with other PTMs, lactylation also directly governs oncogenic protein stability. Lactylation of nucleolar and spindle-associated protein 6 (NOL6) at K54 prevents its ubiquitin-mediated degradation. NOL6 recruits the deubiquitinase STAM binding protein (STAMBP) to stabilize Yin Yang 1 (YY1), enhancing c-Myc transcription and tumor growth. Moreover, a K54-pe4, cell-penetrating peptide inhibitor, could suppress CRC cell proliferation and metastases by inhibiting NOL6 lactylation [140]. The β-catenin signaling pathway is highly activated in colorectal cancer. Lactylation is also involved in the regulation of the β-catenin signaling pathway. Hypoxia-induced lactate accumulation in colorectal cancer contributes to β-catenin lactylation, preventing its phosphorylation-dependent ubiquitination and proteasomal degradation. Stabilized β-catenin accumulates in the nucleus and sustains oncogenic transcriptional programs [141].

#### Ovarian cancer

##### Lactylation and acetylation

In ovarian cancer, malic enzyme 2 (ME2) catalyzes the conversion of glutamine-derived malate into pyruvate, thereby increasing lactate production. Acetylation of ME2 at lysine 156, mediated by acetyl-CoA acetyltransferase 1 (ACAT1), further amplifies glutamine-linked lactate biosynthesis. Elevated lactate not only reinforces metabolic reprogramming but also enhances the lactylation and enzymatic activity of homologous recombination repair proteins, strengthening DNA repair and ultimately conferring resistance to chemotherapy. Targeting this metabolic–epigenetic coupling by disrupting ME2 acetylation and lactylation offers a potential therapeutic strategy to impair DNA repair and restore chemosensitivity [142]. Collectively, these studies underscore that acetylation and lactylation are tightly intertwined regulators that coordinately orchestrate tumor metabolism and damage repair, providing new insight for chemotherapy.

#### Bladder cancer

##### Lactylation and acetylation

Therapeutically, targeting metabolic–epigenetic coupling has shown promise in several tumor contexts. In bladder cancer, mannose directly binds to pyruvate kinase M2 (PKM2) and suppresses its catalytic activity, leading to reduced lactate production [143]. The resulting decrease in PKM2 lactylation, accompanied by enhanced acetylation, facilitates its nuclear translocation and activates pyroptosis-associated signaling pathways, thereby exerting antitumor effects.

#### Gastric cancer

##### Lactylation and ubiquitination

In gastric adenocarcinoma, proteasome non-ATPase regulatory subunit 14 (PSMD14) enhances glycolytic flux and lactate accumulation, leading to elevated H3K27la. This histone mark reciprocally upregulates PSMD14 and Sox9 expression, while PSMD14 deubiquitinates 6-Phosphofructo-2-kinase/Fructose-2,6-bisphosphatase 2 (PFKFB2) to further amplify glycolysis, forming a self-reinforcing H3K27la–PSMD14(deubiquitination)–glycolysis axis that contributes to tumor stemness [144]. NOD-like receptor family pyrin domain-containing protein 12 (NLRP12) stabilizes HK2 by blocking Tripartite motif containing 25 (TRIM25)-mediated ubiquitination, leading to metabolic reprogramming toward glycolysis, thereby increasing H3K18la and activating MYC transcription, thereby facilitating malignant progression [145].

In gastric cancer, lactate secreted by cancer-associated fibroblasts elevates H3K18la in tumor cells. H3K18la upregulates the transcription of abnormal spindle microtubule assembly (ASPM) to promote resistance to anti-PD-1. Mechanistically, ASPM interacts with and promotes BUB3 mitotic checkpoint protein (BUB3)-mediated de-ubiquitination of non-SMC condensin I complex subunit G (NCAPG), inhibiting proteasomal degradation. Next, NCAPG stimulates SRC/STAT3 signaling, increases PD-L1 expression, and facilitates immune evasion. A pharmacological inhibitor (Daturilin) targeting NCAPG markedly improves immunotherapeutic efficacy [146].

#### Esophageal cancer

##### Lactylation and ubiquitination

In esophageal squamous cell carcinoma (ESCC), the pseudogene protein disulfide isomerase family A member 3 pseudogene 1 (PDIA3P1) enhances glycolysis by stabilizing glucose transporter 1 (GLUT1) mRNA and disrupting membrane-associated RING-CH 8 (MARCH8)-mediated ubiquitination and proteasomal degradation of HK2. Excessive lactate production increases H4K8la, which contributes to transcription of *bone morphogenetic protein 7 (BMP7)* and accelerates tumor progression [147]. Restoring HK2 ubiquitination and reducing histone lactylation therefore represent promising therapeutic strategies.

### Inflammation, fibrosis, and immune-mediated disorders

#### Inflammation

##### Lactylation and acetylation

**Fig. 6:**
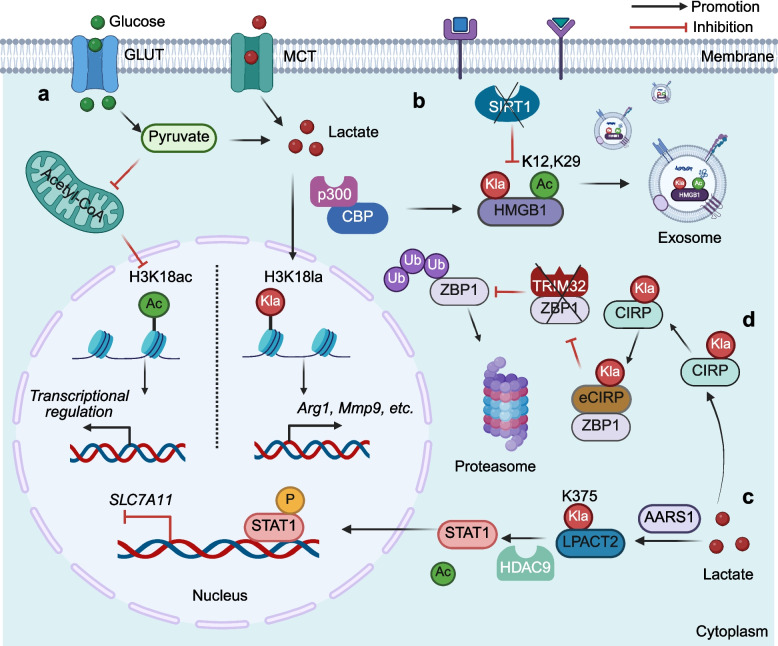
Crosstalk between lactylation and other PTMs in sepsis. **a** Under bacterial infection, increased intracellular lactate triggers p300-mediated upregulation of H3K18la with concurrent inhibition of H3K18ac at the same site. H3K18la alters chromatin structure to promote the expression of *Arg1* and *MMP9*. **b** p300-mediated HMGB1 lactylation and SIRT1-restrained HMGB1 acetylation synergistically drive HMGB1 sorting into exosomes and release into the circulation, which aggravates sepsis progression. **c** AARS1 promotes the lactylation modification of LPACT2, and this suppresses the deacetylation of STAT1 mediated by HDAC9, which in turn leads to elevated STAT1 phosphorylation; phosphorylated STAT1 translocases into the nucleus to inhibit the transcription of *SLC7A11*. **d** CIRP lactylation promotes its binding to ZBP1, which inhibits TRIM33-mediated ubiquitination and degradation of ZBP1, thus aggravating sepsis progression

Lactylation was first characterized on histone in macrophage infection models. In this landmark study, Zhang et al. not only defined this novel modification, but also revealed its inherent crosstalk with the well-characterized acetylation modification [1]. From a metabolic standpoint, lactylation is dependent on lactate as its core substrate, while acetylation uses acetyl-coenzyme A (acetyl-CoA) as its primary acetyl donor, situating the two modifications in separate arms of the cellular metabolic network [48]. The induction of glycolytic metabolic reprogramming, a common event in inflammatory and disease states, is tightly linked to shifts in both lactylation and acetylation levels. For these reasons, in-depth investigation of the crosstalk between acetylation and lactylation is a fundamental and unmet research priority in the field. Upon bacterial infection, the pan lactylation level within macrophages exhibits a persistent upward trend, in contrast to the decreasing pan acetylation level. Moreover, the levels of H3K18la and H3K18ac also display a reciprocal variation tendency, with increased H3K18la marking more M2-specific genes than H3K18ac, suggesting a competitive role of H3K18 modification during macrophage polarization (Fig. 6a) [1]. Another study has also confirmed that H3K18la is elevated while H3K18ac is decreased, and the H3K18la/H3K18ac ratio can serve as a marker for diagnosing the progression of sepsis (Fig. 6a) [148].

Macrophages could uptake circulating lactate through MCTs in sepsis which is associated with the accumulation of intracellular lactate which directly contributes to lactylation of high mobility group box-1 (HMGB1). In addition, lactate could suppress SIRT1 via YAP inactivation and promote the recruitment of p300 to improve the level of HMGB1 acetylation at lysine 12 and 29. Lactylation cooperates with acetylation to promote the release of HMGB1 via exosome secretion, leading to the endothelial barrier dysfunction (Fig. 6b) [149]. These studies strongly indicate that investigating the crosstalk between lactylation and acetylation may provide novel insights into the treatment of infectious inflammation. Although the authors did not identify the lactylation sites of HMGB1 or the modification order between the two modifications, the molecular basis underlying this crosstalk is still largely attributed to alterations in protein spatial conformation and charge distribution.

##### Lactylation and phosphorylation

In sepsis-induced acute lung injury (SI-ALI), elevated glycolysis enhances AARS1-mediated lactylation of LPCAT2 at lysine 375. This modification suppresses STAT1 acetylation while promoting its phosphorylation, nuclear translocation, and transcriptional repression of solute carrier family 7 member 11 (SLC7A11), ultimately triggering ferroptosis in epithelial cells (Fig. 6c) [150].

##### Lactylation and ubiquitination

Lactate accumulation increases the lactylation and release of cold-inducible RNA-binding protein (CIRP), which competitively binds Z-DNA binding protein 1 (ZBP1) and disrupts its interaction with Tripartite motif containing 32 (TRIM32), inhibiting ZBP1 ubiquitination and degradation and contributing to inflammatory damage (Fig. 6d) [151]. As the core scaffold of the PANoptosome, ZBP1 is involved in its assembly and activity regulation [152, 153]. Given that the PANoptosome is a large protein complex, investigating PTMs and the crosstalk among them is of great significance for understanding PANoptosis.

#### Vascular Inflammation

##### Lactylation and phosphorylation

In vascular inflammation, TNF-α first induces phosphorylation of Sox10 via the PI3K/AKT pathway. Subsequently, Sox10 undergoes lactylation in a phosphorylation-dependent manner. Modified Sox10 translocates into the nucleus and promotes the transcription of *Cd74*, *C3*, and *lysozyme 2 (Lyz2)*, promoting vascular smooth muscle cell (VSMC) transdifferentiation and pyroptosis. This process is restrained by G protein signaling 5 (RGS5), which suppresses AKT signaling and Sox10 phosphorylation. These findings suggest that phosphorylation-dependent lactylation of Sox10 modulates inflammatory vascular remodeling [41]. The crosstalk is also attributed to alterations in charge distribution and spatial conformation. Phosphorylation promotes the exposure of lysine residues required for lactylation of Sox10 and modulates its overall charge distribution, thereby facilitating the occurrence of lactylation.

##### Lactylation and ubiquitination

Hypoxia elicits vascular endothelial dysfunction through impaired energy metabolism. Specifically, it drives a metabolic switch from oxidative phosphorylation to glycolysis in vascular endothelial cells, leading to excessive lactate accumulation. This lactate overload triggers lactylation of PKM2; by suppressing PKM2 ubiquitination, this modification enhances the protein’s stability, thereby forming a feedforward regulatory loop that exacerbates mitochondrial dysfunction and vascular endothelial impairment [86]. Targeting glycolysis and PKM2 lactylation, or restoring ubiquitination-dependent PKM2 degradation, is a promising therapeutic approach for relevant diseases. The mechanism underlying this crosstalk is attributed to alterations in lactate levels caused by PTMs of key enzymes in lactate metabolism.

#### Liver fibrosis

##### Lactylation and acetylation

In hepatic fibrosis, direct evidence demonstrates that activation of hexokinase 2 (Hk2) in hepatic stellate cells (HSCs) enhances glycolysis, leading to increased intracellular lactate production. Elevated lactate levels subsequently promote the accumulation of H3K18la, which is preferentially enriched at the promoter regions of fibrosis-related genes such as *Alpha-smooth muscle actin (α-SMA)* and *Collagen type I alpha 1* *(Col1a1)* and facilitates their transcriptional activation. In contrast, genetic ablation of Hk2 or pharmacological inhibition of lactate production markedly suppresses H3K18la accumulation, thereby alleviating hepatic fibrosis. Importantly, modulation of acetylation further highlights the competitive nature of this epigenetic crosstalk. Treatment with HDAC inhibitors significantly ameliorates hepatic fibrosis, accompanied by an increase in H3K18ac and a concomitant decrease in H3K18la. Consistently, lactate supplementation robustly elevates H3K18la levels, whereas acetate treatment suppresses this modification, supporting a competitive relationship between acetylation and lactylation at histone H3 lysine 18 during fibrotic progression [26]. These findings collectively suggest that pharmacological interventions targeting this balance, particularly strategies aimed at enhancing H3K18ac, hold considerable therapeutic potential for hepatic fibrosis.

#### Skin fibrosis

##### Lactylation and phosphorylation

In skin fibrosis, aberrant expression of PKM2 contributes to lactate accumulation, facilitating lactylation of Twist Family BHLH Transcription Factor 1 (Twist1) at lysine 150. This modification enhances Twist1 phosphorylation and nuclear translocation, activating TGF-β signaling and exacerbating fibrosis. Crosstalk between lactylation and phosphorylation coordinately regulates Twist1 to promote fibrotic progression. Thus, targeting either lactate generation and lactylation, or Twist1 phosphorylation, represents a promising therapeutic strategy for skin fibrosis [154]. While individual PTMs can modulate protein function, the combination of these two PTMs and their functional crosstalk may exert a far more potent regulatory effect on protein activity and biological function. Moreover, the basis for this crosstalk may lie in changes to local electrostatic charge or steric hindrance at the modified protein residues, while the full spectrum of regulatory pathways awaits further in-depth investigation.

#### Lupus nephritis

##### Lactylation and ubiquitination

Lupus nephritis (LN) represents a severe complication of systemic lupus erythematosus (SLE). The de novo generation of these pathogenic autoantibodies is tightly governed by follicular helper T cells (Tfh cells) residing in secondary lymphoid organs. Notably, elevated lactate concentrations promote the PCAF-mediated lactylation of B-cell lymphoma 6 protein (BCL6) protein; this post-translational modification subsequently triggers K6 and K29-linked ubiquitination at lysine 430 of BCL6, thereby abrogating its proteasomal degradation. Sustained BCL6 expression ultimately amplifies Tfh cell differentiation and aggravates autoimmune-mediated renal injury in LN [68]. Another study reported that DNA-containing immune complexes (DNA-ICs), a key pathogenic factor in SLE, induce elevated lactate levels, which in turn enhance both lactylation and ubiquitination of pre-B-cell leukemia transcription factor 1 (PBX1). This post-translational modification profile contributes to PBX1 degradation by TRIM21, thereby accelerating disease progression. Pharmacological inhibition of lactylation and ubiquitination-mediated BCL6 and PBX1 degradation holds substantial therapeutic potential for the management of LN [155].

#### Rheumatoid Arthritis

##### Lactylation and acetylation

Downregulation of RNA-binding motif protein 25 (RBM25) is associated with the accumulation of non-lactylated short isoform of ATP citrate lyase (AclyL), H3K9ac and H3K18ac. These factors have been implicated in the occurrence and development of rheumatoid arthritis [156].

#### Endometriosis

##### Lactylation and SUMOylation

Endometriosis is a gynecological disease defined by the growth of endometrium-like tissues within and beyond the pelvic cavity [157]. Studies have confirmed lactate accumulation in endometriosis. Mechanistically, histone lactylation at H3K18 contributes to transcription of *RASD family member 2 (RASD2)*, which upregulates an E3 ligase and enhances SUMOylation of CTP Synthase 1 (CTPS1), stabilizing the protein. Therefore, H3K18la promotes the SUMOylation of CTPS1 and aggravates onset of the disease [158]. Moreover, 2-DG-mediated glycolysis inhibition ameliorates the disease.

### Neurodegenerative and neurological disorders

#### Alzheimer’s disease

##### Lactylation and acetylation

**Fig. 7:**
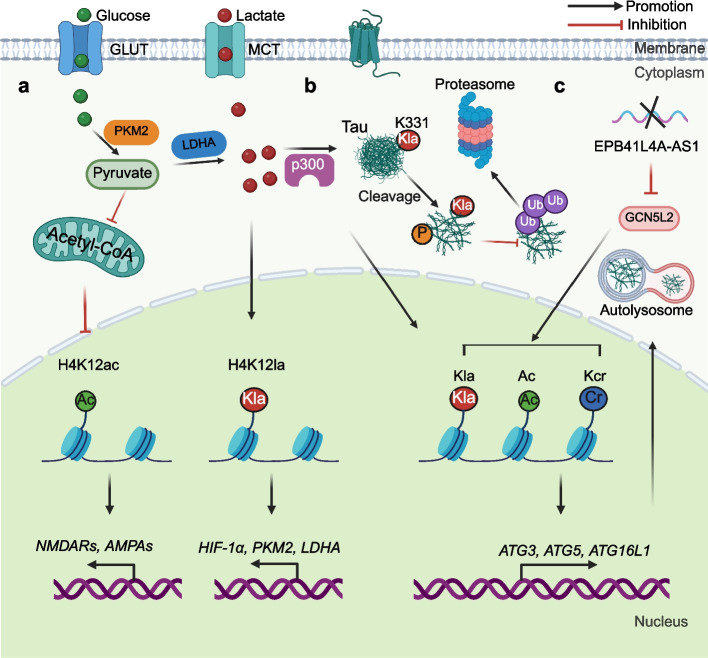
Crosstalk between lactylation and other PTMs in Alzheimer’s disease. **a** In Alzheimer’s disease, reduced H4K12ac and increased H4K12la downregulate the transcription of *NMDARs* and *AMPAs*, and upregulate the expression of *HIF-1α*, *PKM2* and *LDHA*, respectively; the two alterations synergistically aggravate AD progression. **b** Tau K331la drives sequential tau cleavage and phosphorylation, inhibits its ubiquitination-dependent proteasomal degradation, and promotes pathological tau aggregation in Alzheimer’s disease. **c** EPB41L4A-AS1 reduces GCN5L2 expression, lowering histone lactylation, ubiquitination, and crotonylation. These modifications jointly remodel chromatin, inhibit *ATG3/ATG5/ATG16L1* expression, weaken autophagy, and reduce autophagic clearance of tau protein

The H4K12la level was elevated in the microglia adjacent to Aβ plaques within the Alzheimer’s disease (AD) mouse model (5XFAD) and AD patients. Moreover, it was substantially enriched in the promoter regions of glycolysis genes, which have been implicated in subsequent transcriptional activation and the progression of AD [159]. Another study demonstrated that the levels of H4K12ac were downregulated in AD and 5XFAD mouse model thus leading to a diminished enrichment within the promoter regions of *N-Methyl-D-aspartate receptors (NMDARs)* and *α-Amino-3-hydroxy-5-methyl-4-isoxazolepropionic acid receptors (AMPARs)* reducing their expression. Additionally, administration of acetate was capable of upregulating the levels of H4K12ac and thereby leading to a further augmentation in the expression of NMDARs and AMPARs, consequently enhancing synaptic plasticity and cognitive function within 5XFAD mice [160]. The above research may indicate that there is a competitive relationship between acetylation and lactylation on the H4K12 site, which jointly regulates AD progression (Fig. 7a). Therefore, shifting the H4K12 modification balance toward H4K12ac or suppressing H4K12la offers a novel and effective therapeutic avenue for AD.

##### Lactylation and phosphorylation

Tau aggregation represents a central pathological hallmark in the progression of AD. Tau lactylation is markedly elevated in AD brain tissues, with lysine 331 identified as a key modification site. Lactylation contributes to tau phosphorylation and proteolytic cleavage while inhibiting ubiquitination-mediated degradation, thereby accelerating tau accumulation and disease progression (Fig. 7b) [161]. Accordingly, targeting tau lactylation to restore proteostatic balance represents a potential therapeutic avenue.

##### Lactylation and crotonylation

In Alzheimer’s disease (AD), the downregulation of the histone acetyltransferase general control nonderepressible 5-like 2 (GCN5L2) leads to a concurrent decrease in histone crotonylation and lactylation. This dual reduction reflects a breakdown in their cooperative crosstalk, which impairs the expression of autophagy-related genes and is associated with the accumulation of β-amyloid (Aβ) plaques (Fig. 7c) [162]. To date, documented cooperative crosstalk between lactylation and crotonylation remains largely confined to histones, likely due to their relatively low abundance in non-histone contexts. Nevertheless, a deeper understanding of their integrated roles in physiology and disease requires elucidating the precise mechanisms of their cooperative interplay, which may involve mutual reinforcement, sequential activation, or the coordinated recruitment of effector proteins.

#### Parkinson’s disease

##### Lactylation and acetylation

Glucose metabolism is significantly enhanced during the progression of PD, accompanied by excessive lactate accumulation and a marked increase in H3K9la. Meanwhile, H3K9ac levels are decreased [163]. In the motor cortex, the total histone acetylation level was increased, and there was a net increase in histone H3 acetylation due to increased H3K14ac and H3K18ac. These findings suggest that acetylation and lactylation on histones may act antagonistically to regulate PD progression [164]. We should simultaneously focus on the changes in histone acetylation and lactylation levels in PD to make a better understanding of the underlying mechanism.

#### Neuropathic pain

##### Lactylation and phosphorylation

In neuropathic pain, nerve injury induces aberrant phosphorylation of Sox9, a transcription factor selectively expressed in astrocytes. Phosphorylated Sox9 activates transcription of HK1, enhancing glycolytic flux and promoting accumulation of H3K9la. H3K9la promotes the expression of *Glial fibrillary acidic protein (Gfap), Complement factor B (Cfb), C3, Jun, C–C motif ligand 2 (Ccl2), and Cxcl2*, drives the transition of astrocytes toward a pathological phenotype, and mediates the development of pain hypersensitivity [165]. The Sox9–HK1–H3K9la axis thus represents a critical molecular pathway in neuropathic pain pathogenesis, and inhibition of Sox9 phosphorylation or lactate production may offer therapeutic benefit.

The crosstalk arises because the phosphorylated transcription factor Sox9 activates the expression of HK1, leading to elevated lactate levels and increased histone lactylation. This is analogous to the scenario in which LDHA and PKM2 phosphorylation enhances lactate metabolism and thereby promotes lactylation. Therefore, whether increased lactate metabolism occurs at the transcriptional level or via post‑translational modifications, it is essential to examine whether a crosstalk exists with lactylation and other PTMs.

### Metabolic-associated diseases and organ injury

#### Metabolic dysfunction-associated fatty liver disease

##### Lactylation and acetylation

Dysregulated histone lactylation and acetylation are also implicated in Metabolic dysfunction-associated fatty liver (MASLD). Notably, the concurrent accumulation of H3K18la and H3K27ac has been identified in MASLD [166, 167]. However, the gene expression regulated by H3K27ac and H3K18la is distinct: H3K27ac regulates the expression of *the enzyme tryptophan 2,3-dioxygenase (TDO2)* [166], while H3K18la regulates the expression of *Hypoxia-inducible factor 1 (HIF1-α)* [167]*.* This suggests that lactylation and acetylation may act cooperatively and have been implicated in disease progression by facilitating the transcription of distinct but complementary gene sets.

##### Lactylation and ubiquitination

Under physiological conditions, YTHDC1 protects against MASLD by inhibiting Protein Tyrosine Phosphatase Non-Receptor Type 2 (PTPN22)-mediated dephosphorylation and activation of NLR Family Pyrin Domain Containing 3 (NLRP3), ultimately promoting the release of IL-1β and IL-18. However, in both MASLD patients and corresponding mouse models, expression of YTH domain-containing protein 1 (YTHDC1) is significantly reduced. Mechanistically, lactate accumulation leads to AARS1-mediated lactylation specifically at lysine 565 (K565), which facilitates ubiquitination-dependent degradation of YTHDC1. Loss of YTHDC1 therefore removes this protective checkpoint, exacerbating disease pathology. Notably, treatment with mebendazole, a small-molecule agent targeting YTHDC1, effectively restores its activity and alleviates MASLD-associated pathological features [168].

#### Liver injury

##### Lactylation and ubiquitination

Moreover, previous studies have established that lactate derived from the microenvironment of acetaminophen-induced acute liver injury upregulates Caspase-11 expression, potentiates gasdermin D (GSDMD) activation, and accelerates macrophage pyroptosis to exacerbate liver injury. This process is governed by lactate-promoted neural precursor cell expressed, developmentally down-regulated 4 (NEDD4) lactylation, which impairs NEDD4-Caspase-11 interaction, blocks Caspase-11 ubiquitin-dependent degradation, and thereby facilitates cellular pyroptosis; notably, suppressing lactate accumulation and NEDD4 lactylation constitutes an effective intervention to alleviate such liver injury [87].

#### Kidney injury

##### Lactylation and ubiquitination

Lactylation is increased in kidney tissues of acute kidney injury (AKI) patients and mice. Lactylation of Aldehyde dehydrogenase 2 family member (ALDH2) at lysine 52 inhibits the interaction with Prohibitin 2 (PHB2). This is associated with an increase in free PHB2 and enhances ubiquitin-mediated degradation of PHB2, impairing mitophagy and worsening mitochondrial dysfunction, which ultimately accelerates disease progression [169]. The molecular basis underlying this crosstalk is consistent with the aforementioned ZBP1 case: one PTM leads to the occupation or increased release of substrates for lactylation, which constitutes the molecular basis for the crosstalk between lactylation and other PTMs.

#### Allograft injury

##### Lactylation and ubiquitination

Nucleophosmin 1 (NPM1), an epigenetic regulator, is subjected to lactylation at lysine 257, a modification catalyzed by the lactyltransferase AARS1. Mechanistically, this post-translational modification preserves NPM1 protein stability by attenuating Murine Double Minute 2 (MDM2)-mediated ubiquitination, while concurrently reinforcing transcriptional repression of SLC7A11. Collectively, these alterations are associated with ferroptosis, aggravated allograft injury, and delayed functional recovery of the transplanted kidney [170].

### Aging-associated and cardiovascular degenerative diseases

#### Intervertebral disc degeneration

##### Lactylation and phosphorylation

In intervertebral disc degeneration (IDD), metabolic imbalance is closely linked to aberrant lactylation–phosphorylation signaling. Degenerated nucleus pulposus tissues exhibit reduced glutamine levels alongside elevated lactate accumulation and lactylation of AMP-activated protein kinase alpha subunit (AMPKα). Glutamine supplementation suppresses glycolysis, reduces lactate production, downregulates lactylation of AMPKα, and enhances its phosphorylation. These coordinated changes are associated with reduced cellular senescence, enhanced autophagy, and partial restoration of extracellular matrix synthesis. Inhibition of glycolysis alone similarly reduces lactylation levels and is associated with a delay in IDD progression, highlighting metabolic–epigenetic coupling as a potential therapeutic target [171].

#### Atherosclerosis

##### Lactylation and methylation

Exercise‑induced lactylation of methyl‑CpG‑binding protein 2 (MeCP2) at lysine 271 directly cooperates with H3K36me3 to suppress the expression of runt‑related transcription factor 1 (RUNX1). This combined epigenetic regulation contributes to M2 polarization of macrophages, thereby accelerating stable plaque formation and exacerbating atherosclerosis [172].

#### Myocardial aging

##### Lactylation and methylation

H3K18la enhances local chromatin accessibility and upregulates inflammatory gene expression, the accumulation of H3K27me3 counteracts this process and delays the onset of aging‑related cardiac decline [29]. Proteomic analyses also indicate that histones possess overlapping residues susceptible to both methylation and lactylation. Therefore, the balance between histone lactylation and methylation may be crucial for maintaining healthy cardiac function.

## Methodological and analytical advances

Accurate detection, confident site assignment, and reliable quantification of protein lactylation are prerequisites for elucidating its distribution, dynamics, and biological significance. As a recently identified lysine acylation with strong structural similarity to acetylation, crotonylation, and other short-chain acyl modifications, lactylation poses distinct methodological and analytical challenges [1, 15, 173–175]. Importantly, many experimental platforms used to detect lactylation are shared with those for other PTMs, raising critical issues related to specificity, cross-reactivity, and interpretability [15, 175]. Therefore, a unified methodological framework integrating biochemical, proteomic, metabolic, and functional approaches is essential for robust characterization of lactylation and its crosstalk with other PTMs.

### Antibody-based and affinity-based detection of lactylation

Antibody-based approaches remain the most widely used methods for assessing protein lactylation due to their accessibility, scalability, and compatibility with routine laboratory techniques [1, 15]. Pan-anti-lactyl lysine antibodies enable rapid evaluation of global lactylation levels using western blotting, immunofluorescence, immunohistochemistry, and enzyme-linked immunosorbent assay, providing an overview of lactylation dynamics across experimental conditions, tissues, and disease states [1, 12, 175, 176].

Site-specific lactylation antibodies further allow interrogation of individual modification events with defined functional relevance and are particularly valuable for validating mass spectrometry–identified sites [177–181]. Importantly, antibody-based assays can be readily combined with antibodies recognizing other PTMs, such as acetylation, ubiquitination, and SUMOylation—enabling parallel or sequential detection of multiple modifications within the same biological sample [81].

However, the high structural similarity between lactylation and other lysine acylations introduces a major technical limitation: antibody cross-reactivity. Signals attributed to lactylation may partially reflect acetylation or crotonylation, especially in metabolically active tissues. Rigorous validation is therefore essential and should include peptide competition assays, testing against structurally related acyl modifications, and confirmation using modification-deficient mutants. Without such controls, antibody-based measurements may overestimate or misinterpret true lactylation abundance [81].

### Mass spectrometry–based proteomics and quantitative strategies

High-resolution liquid chromatography–tandem mass spectrometry (LC–MS/MS) currently represents the gold standard for unbiased identification and quantification of lactylation sites. Advances in instrumentation, enrichment strategies, and data analysis pipelines have greatly expanded the catalog of lactylated proteins across diverse biological systems [1, 173, 174, 182].

Because lactylation often occurs at low stoichiometry, selective enrichment is typically required prior to MS analysis. Immunoaffinity purification using pan-anti-lactyl lysine antibodies is the most commonly employed approach, while chemical derivatization and acyl-specific labeling strategies have also been explored. Quantitative proteomic workflows—including SILAC, tandem mass tag (TMT) labeling, and label-free quantification—enable comparative analyses of lactylation dynamics in response to metabolic perturbations or disease progression [1, 173, 178, 183].

Despite these advances, MS-based analysis faces inherent challenges. These include false-positive identifications caused by ambiguous spectra, isobaric or near-isobaric interference from other acyl modifications, and limited information on site-specific stoichiometry. Moreover, because multiple PTMs may coexist on the same lysine or protein, confident discrimination between competing modifications requires stringent false discovery rate control, manual spectral inspection, and complementary validation strategies [175, 184].

### Metabolic tracing and cross-PTM discrimination

Metabolic tracing using stable isotope–labeled lactate (e.g., ^13C-lactate) provides a powerful approach to directly link lactate metabolism with protein lactylation. Detection of isotopically labeled lactyl groups on peptides offers definitive evidence for the metabolic origin of the modification and facilitates discrimination between enzymatic and non-enzymatic lactylation pathways [15].

When combined with LC–MS/MS, isotope tracing enables precise mapping of lactylation sites derived from specific lactate pools and allows temporal analysis of modification dynamic [1]. Importantly, this strategy can be integrated with simultaneous analysis of other PTMs within the same sample, providing a means to investigate how metabolic flux differentially shapes lactylation, acetylation, and phosphorylation. Nevertheless, careful optimization of labeling duration and concentration is required to avoid perturbing cellular metabolism and confounding downstream interpretations [81, 173, 185, 186].

### Methodological limitations and best-practice validation

Despite rapid technical progress, several methodological and analytical limitations continue to constrain lactylation research. First, the chemical similarity between lactylation and other lysine acylations increases the risk of misannotation, particularly in large-scale proteomic datasets. Second, co-occurrence of multiple PTMs on the same lysine residue complicates enrichment and detection, potentially biasing modification-specific measurements. Third, accurate quantification of site-specific lactylation stoichiometry remains technically challenging, limiting direct assessment of functional dominance [15, 81, 178].

To address these issues, rigorous validation practices are essential. Recommended strategies include the use of synthetic lactylated peptides to benchmark MS performance and antibody specificity, application of orthogonal antibodies recognizing distinct modification chemistries, and site-directed mutagenesis to confirm lactylation-dependent functional effects. Integration of biochemical assays, proteomics, and functional perturbation experiments substantially enhances confidence in lactylation assignments and mechanistic conclusions [81, 173, 187, 188].

### Emerging and integrative methodologies

Beyond conventional approaches, emerging techniques such as proximity labeling, chemical proteomics, and structural biology are beginning to provide new insights into lactylation-mediated protein–protein interactions and conformational regulation [52, 77, 189–191]. In addition, single-cell and spatially resolved proteomic technologies hold promise for capturing lactylation heterogeneity within complex tissues and microenvironments, an aspect that is particularly relevant for studying metabolic diseases, inflammation, and cancer [192–195]. Subcellular fractionation to separate proteins followed by compartment-specific mapping of lactylated peptides in a model cell line has emerged as an effective strategy to overcome the limitations of whole-cell lysate–based enrichment [196]. By enabling spatially resolved lactylome profiling, this method facilitates the discovery of low-abundance Kla sites and supports the analysis of subcellularly regulated PTM crosstalk.

Collectively, the integration of antibody-based detection, quantitative proteomics, metabolic tracing, and rigorous validation constitutes a comprehensive toolkit for studying protein lactylation. Continued methodological refinement, together with transparent reporting of technical limitations, will be critical for improving specificity, reproducibility, and mechanistic resolution, thereby enabling a deeper understanding of lactylation biology and its crosstalk with other post-translational modifications.

## Targeting the crosstalk between lactylation and other PTMs: therapeutic potential and future directions

PTMs are fundamental mechanisms for regulating protein function, localization, stability, and interactions [197–199]. The discovery of lysine lactylation (Kla) has transformed lactate from a metabolic byproduct into a key signaling molecule that directly links cellular metabolic status to epigenetic and proteomic regulation. As detailed throughout this review, lactylation functions as a pivotal regulator at the interface between health and disease [1, 15]. Crucially, its biological impact is rarely exerted in isolation but is predominantly realized through a complex network of cooperative and competitive interactions with other major PTMs, including acetylation, phosphorylation, ubiquitination/SUMOylation, crotonylation, and methylation. This crosstalk, occurring on both histone and non-histone proteins, fine-tunes critical cellular processes such as gene transcription, signal transduction, protein degradation, and metabolic adaptation [26, 28, 30, 31]. Consequently, dysregulation of this interconnected modification network is a hallmark of numerous diseases, particularly cancer, metabolic disorders, fibrosis, and neurodegeneration, making it a compelling frontier for therapeutic intervention.

### Molecular logic and therapeutic implications of the crosstalk network

The preceding sections systematically delineate the multifaceted crosstalk between lactylation and other PTMs, revealing underlying patterns with direct therapeutic relevance.

#### Shared infrastructure drives competition and cooperation

A central theme is the shared molecular machinery between lactylation and other acylations, especially acetylation. Common “writer” enzymes (e.g., p300/CBP), “eraser” enzymes (e.g., HDACs, Sirtuins), and potentially overlapping “readers” create a direct molecular interface for competition at identical lysine residues (e.g., H3K18, p53 K120) [8, 15, 49]. The outcome at these “modification hotspots” is dynamically determined by the local concentration of metabolic precursors (lactate vs. acetyl-CoA) and the activity of bifunctional enzymes [48]. This competition can dictate cell fate, as seen in macrophage polarization (H3K18la vs. H3K18ac) or liver fibrosis progression [1, 26].

#### Sequential and synergistic regulation enables precision

Beyond competition, sequential and synergistic crosstalk forms sophisticated regulatory circuits. Phosphorylation often serves as a priming event for subsequent lactylation (e.g., Sox10 in vascular inflammation), creating a dependency that integrates external signals with metabolic state [41]. Cooperative actions, such as the combined effect of H3K27ac and H3K18la in cellular reprogramming or H3K9cr and H3K9la in delaying Alzheimer’s pathology, demonstrate how modification combinations produce distinct biological outputs that cannot be achieved by single PTMs [31, 35].

#### Crosstalk as a metabolic-epigenetic- signaling nexus

The interconnection between lactylation and other PTMs, particularly phosphorylation and ubiquitination, establishes powerful feedback loops that amplify disease states. For instance, in glioma, lactate-driven histone lactylation upregulates LINC01127, which enhances JNK phosphorylation, further boosting glycolysis and lactylation—a self-reinforcing cycle promoting tumor growth. Similarly, lactylation can antagonize ubiquitin-mediated degradation (e.g., TFEB, β-catenin, mutant RHOA), stabilizing oncoproteins and driving tumorigenesis [28, 124, 133, 141].

### Strategic interventions targeting the crosstalk network

The mechanistic understanding of this crosstalk network reveals multiple nodes for therapeutic intervention, moving beyond merely targeting lactate production to more precise strategies.

#### Metabolic modulation at the source

Inhibiting glycolysis or lactate production (e.g., using LDHi, Shikonin) reduces the global substrate pool for lactylation, thereby dampening the entire lactylation-driven crosstalk network [54, 200]. This approach has shown efficacy, such as in reversing chemoresistance by reducing MRE11 lactylation. However, its specificity is low, as it broadly affects many lactate-dependent processes, potentially leading to significant side effects [27, 201–204].

#### Precision targeting of key nodes

A more refined strategy involves directly interfering with specific lactylation events or their functional interfaces within the crosstalk network. This can be achieved through the following approaches. (1) Small-Molecule Inhibitors: Developing compounds that block the activity of specific lactyltransferases (e.g., targeting p300’s lactyltransferase activity) or disrupt the binding of “reader” proteins to lactylated marks [26, 80, 204]. (2) Peptide-based Therapeutics: Utilizing cell-penetrating peptides (e.g., K54-pe4) designed to mimic or block specific lactylation sites, thereby preventing pathogenic protein–protein interactions (e.g., disrupting NOL6 lactylation to inhibit YY1 stabilization in colorectal cancer) [140]. (3) PROTAC and Molecular Glues: Employing proteolysis-targeting chimeras (PROTACs) to degrade key enzymes in the crosstalk network (e.g., ACAT2 in pancreatic cancer) or using molecular glues to modulate complex formation [5, 205].

#### Restoring homeostatic balance

In cases of competitive crosstalk, therapeutic aim can be to restore a protective modification. For example, in liver fibrosis, class I HDAC inhibitors increase H3K18ac, which competitively reduces pathogenic H3K18la, thereby ameliorating disease.

Among these, precision-targeting strategies (Strategy 2) are preferable from a therapeutic development perspective, as they offer the potential for higher specificity, reduced off-target effects, and the ability to correct specific pathological circuits without globally disrupting metabolism.

## Discussion and future perspectives

In conclusion, lactylation has emerged as a critical PTM that functions as a metabolic sensor, translating fluctuations in lactate—a hallmark of activated glycolysis in many diseases—into precise regulatory signals. Its true biological significance, however, is embedded within its extensive crosstalk with the existing PTM landscape. This review has synthesized evidence demonstrating that through competitive occupancy, sequential priming, and cooperative action, lactylation interfaces with acetylation, phosphorylation, ubiquitination/SUMOylation, and other modifications to govern gene expression, protein function, and cellular destiny in health and disease (Table 1).

**Table 1:** Case studies on the crosstalk between lactylation and other Post-translational Modifications (PTMs) in health and disease treatment interventions

Classification	Biological processes	Targets of lactylation	Targets of other PTMs	Relationship	Inhibitors	Refs
Lactylation and Acetylation	Stem Cell Maintenance and Differentiation	H3K18la	H3K27ac	Coordination	/	[35]
	Hematopoiesis	HDAC1(K412, K452)	H3K27ac	Coordination	/	[94]
	Osteogenic Differentiation	H3K18la	H3K18ac	Coordination	/	[95, 96]
	Neurite Outgrowth	α-tubulin(K40)	α-tubulin(K40ac)	Competition	/	[74]
	Meiosis	H3K18la H4K12la	H4K12ac, H4K16ac	Coordination	/	[92]
	Hepatocellular Carcinoma	H3K56la	PDHX(K488ac)	Competition	/	[112]
	Pancreatic Cancer	H3K18la	MTCH2	Coordination	AP1	[5]
	Glioma	H4K16la	LMNA(K470)	Coordination	/	[130]
		H3K18la	GTPSCS (K73ac)	Coordination	/	[64]
	Ovarian Cancer	NBS1, MRE11	ME2(k156ac)	Coordination	K604	[142]
	Bladder Cancer	PKM2	PKM2	Competition	Mannose	[143]
	Inflammation	H3K18la	H3K18ac	Competition	/	[1, 148]
		HMGB1	HMGB1 (K12ac, K29ac)	Coordination	/	[149]
	Hepatic Fibrosis	H3K18la	H3K18ac	Competition	MS275	[26]
	Alzheimer’s disease	H4K12la	H4K12ac	Competition	/	[159, 160]
	Metabolic Dysfunction-associated Steatotic Liver Disease	H3K18la	H3K27ac	Coordination	/	[166, 167]
Lactylation and Phosphorylation	Folliculogenesis	CREB(K36)	CREB(Ser136)	Coordination	/	[38]
	Muscle Homeostasis	VSP34(k356, K781)	LDHA(Ser196)	Coordination	/	[32, 101]
	Hepatocellular Carcinoma	H3K18la	YBX1(Ser102)	Coordination	/	[113]
		TPX2(K249)	AURKA(Thr288)	Coordination	GSK2837808A	[114]
		YAP(K102)	YAP(Ser127)	Competition		[115]
		H3K18la	STAT3	Coordination	/	[116]
	Pancreatic Ductal Adenocarcinoma	H3K18la	LDHA(Try239)	Coordination	/	[122]
	Breast Cancer	MRE11(K673)	CBP	Coordination	LDHi,CBPi	[27]
	Glioma	Histone	JNK	Coordination	/	[133]
	Lung Cancer	cGAS(K21, K415)	PSMA4(Ser188)	Competition	FX11	[136]
	Colorectal Cancer	XLF(K288)	GCN5	Coordination	laX-KBM GCN5i	[139]
	Inflammation	LPCAT2(K175)	STAT1	Coordination	/	[150]
		SOX10	SOX10(Ser24)	Coordination	/	[41]
	Skin Fibrosis	Twist1(K150)	Twist1(Ser68)	Coordination	/	[154]
	Alzheimer’s Disease	Tau(K331)	Tau (Ser199, Thr205, Thr217)	Competition	/	[161]
	Neuropathic pain	H3K9la	Sox9 (Ser181)	Coordination	/	[165]
	Intervertebral Disc Degeneration	AMPKα	AMPKα	Competition	Glutamine	[171]
Lactylation and Ubiquitination	Hepatocellular Carcinoma	H3K18la	KEAP1	Coordination	/	[118]
		H3K18la	PD-L1	Coordination	OXA	[119]
	Pancreatic Cancer	TFEB(K91)	TFEB	Competition	/	[28]
	Breast Cancer	RHOA (K118, K162)	RHOA	Competition	Sodium Oxamate, Y-27632	[124]
		BLM(K24)	BLM	Competition	/	[125]
	Lung Cancer	H3K18la	p21	Coordination	/	[137]
	Colorectal Cancer	NOL6(K56)	YY1(K339)	Coordination	K54-pe4	[140]
		β-catenin	β-catenin	Competition	/	[141]
	Gastric Cancer	H3K27la	PFKFB2(K355)	Coordination	Daclatasvir	[144]
		H3K18la	HK2	Coordination	/	[145]
		H3K18la	NCAPG	Coordination	Daturilin	[146]
	Esophageal Cancer	H4K8la	HK2	Coordination	/	[147]
	Spesis	CIRB	ZBP1	Competition	/	[151]
	Inflammation	PKM2	PKM2	Competition	/	[86]
	Lupus Nephritis	BCL6	BCL6(K430)	Coordination	/	[68, 155]
	Metabolic Dysfunction-associated Steatotic Liver Disease	YTHDC1(K565)	YTHDC1	Coordination	Mebendazole	[168]
	Liver Injury	NNDD4	Caspase-11	Competition	/	[87]
	Acute Kidney Injury	ALDH2	PHB2	Coordination	/	[169]
	Delayed Graft Function	NPM1(K257)	NPM1	Coordination	/	[170]
Lactylation and SUMOylation	Breast Cancer	ZMIZ1(K834)	ZMIZ1	Competition	/	[127]
	Lung Cancer	SUMO(K11)	ACSL4	Coordination	Peptide (K11R-Pep)	[30]
	Endometriosis	H3K18la	CTPS1	Coordination	/	[158]
Lactylation and Crotonylation	Neural Development	H3K18la	H3K9cr	Coordination	MS-275	[31]
	Alzheimer’s disease	Histone	Histone	Coordination	/	[162]
Lactylation and Methylation	Breast Cancer	PRMT1(K134, K145)	Vimentin(R64)	Coordination	MS023	[128]
	Atherosclerosis	MeCP2(K271)	H3K36me3	Coordination	/	[172]
	Myocardial Aging	H3K18la	H3K27me3	Competition	/	[29]

When two modifications synergistically promote the progression of both physiological homeostasis or pathological processes, they are regarded as coordination; when their functions are antagonistic, they are considered competition

Targeting this crosstalk network holds immense therapeutic promise but requires navigating its complexity. Future research must advance on several fronts: (1) Technology Development: There is a pressing need for tools with higher specificity to map the “multimodificome”—simultaneously quantifying multiple PTMs on the same protein or even the same residue in a dynamic manner. Advanced mass spectrometry methods, more specific antibodies, and single-cell epigenomic/proteomic techniques will be crucial. (2) Mechanistic Depth: Structural biology studies are essential to elucidate how different modification combinations (e.g., lactylation + phosphorylation) alter protein conformation and interaction interfaces. Furthermore, the systematic identification and validation of dedicated “readers” for lactylated non-histone proteins remain a significant gap. (3) Systems-Level Understanding: Computational modeling and AI-based prediction should be integrated with multi-omics data to map disease-specific PTM interaction networks, predict key regulatory nodes, and identify optimal therapeutic targets. (4) Translational Exploration: The promising preclinical examples of targeting lactylation crosstalk (e.g., in fibrosis, cancer drug resistance) must be rigorously translated. This includes developing pharmacologically optimized compounds, evaluating their efficacy and safety in advanced disease models, and ultimately designing clinical trials that consider patient stratification based on metabolic and PTM signatures.

**Fig. 8:**
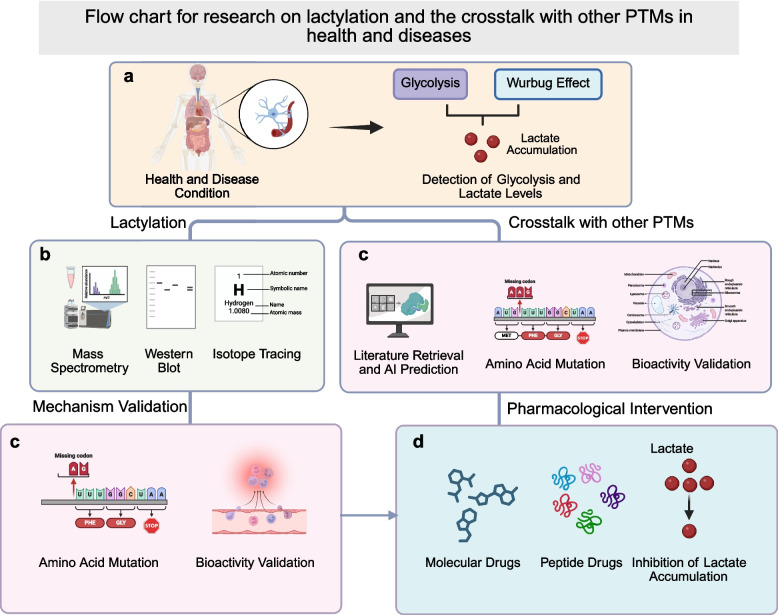
Flow chart for research on lactylation and the crosstalk with other PTMs in health and disease. **a** Warburg effect and glycolysis activation lead to lactate accumulation, which may contribute to an increase in lactylation. **b** Mass spectrometry, antibody-specific Western blot, isotope labeling, and other approaches may facilitate the discovery of lactylation. **c** Amino acid site-directed mutagenesis and phenotypic analysis should be applied to verify the function of lactylation and its specific modification sites. A literature review is preferable for investigating crosstalk, given that crosstalk may exert a more significant impact on the key sites of proteins. Mass spectrometry and AI-based prediction methods can be used as supplementary approaches. **d** Intervention in lactylation using small-molecule or peptide drugs, or inhibition of glycolysis with agents such as Shikonin, can achieve the goal of disease treatment*. Note: The former is preferable, as the latter is associated with poor specificity and tends to induce more severe side effects*

Ultimately, the study of lactylation and its crosstalk represents a paradigm shift in understanding how metabolism, epigenetics, and cellular signaling are inseparably linked. By moving from viewing PTMs in isolation to deciphering their interconnected language, we gain not only a deeper comprehension of fundamental biology but also a powerful new framework for diagnosing and treating a wide array of human diseases (Fig. 8). The journey from characterizing this novel modification to exploiting its therapeutic potential is well underway, offering a promising path toward more precise and effective medicine.

## Data Availability

The data that support the findings of this study are available from the corresponding author upon reasonable request.
